# Viral infection and brain inflammation with seizures in PARK7 deficiency

**DOI:** 10.70962/jhi.20250044

**Published:** 2025-12-26

**Authors:** Jonas Lønskov, Annika Sünderhauf, Sisse Andersen, Caroline Bækmann Jeppesen, Franziska Winzig, Daniëla Maria Hinke, Johanna L. Heinz, Kenneth Thomsen, Mette B. Thorup, Thomas Zillinger, Bettina Bundgaard, Kerstin De Keukeleere, Sofie Eg Jørgensen, Jakob Ek, Elsebet Østergaard, Jakob Christensen, Mette Møller Handrup, Renee M. van der Sluis, Trine H. Mogensen

**Affiliations:** 1 Department of Biomedicine, Aarhus University, Aarhus, Denmark; 2 Center for Immunology of Viral Infections, Aarhus University, Aarhus, Denmark; 3 Department of Clinical Medicine, Aarhus University, Aarhus, Denmark; 4 Department of Pediatrics, Rare Diseases, Aarhus University Hospital, Aarhus, Denmark; 5 Department of Dermatology and Venereology, Aarhus University Hospital, Aarhus, Denmark; 6 Department of Radiology, Aarhus University Hospital, Aarhus, Denmark; 7 Institute of Clinical Chemistry and Clinical Pharmacology, University Hospital Bonn, Bonn, Germany; 8 Department of Clinical Genetics, Rigshospitalet, Copenhagen, Denmark; 9 Department of Clinical Medicine, University of Copenhagen, Copenhagen, Denmark; 10 Department of Neurology, Aarhus University Hospital, Aarhus, Denmark; 11 Department of Infectious Diseases, Aarhus University Hospital, Aarhus, Denmark

## Abstract

Respiratory syncytial virus (RSV) is a major health problem worldwide, particularly in infants and young children. The infection can progress to life-threatening lower respiratory disease and, in rare cases, involves the central nervous system. We explore the pathophysiology in a child with high fever, seizures, and encephalopathy with brain inflammation during severe RSV infection. Whole-genome sequencing revealed homozygosity for a rare loss-of-function variant in the early-onset Parkinson-related gene *PARK7*/*DJ-1*. PARK7 plays a role in immune regulation, stress responses, and cell death. The patient’s Peripheral blood mononuclear cells and fibroblasts exhibited increased inflammatory cytokine responses, impaired RSV-induced apoptosis, and dampened autophagy. Studies in PARK7-deficient neuronal cells recapitulated the patient’s cellular phenotype, which was reversed upon PARK7 reconstitution. To our knowledge, this is the first association between PARK7 deficiency and RSV-induced brain inflammation, encephalopathy, and seizures. Collectively, our results demonstrate a role for PARK7 in regulation of inflammation and cellular homeostasis and suggest that PARK7 deficiency may aggravate infectious disease and cause immunopathology.

## Teaser

Here, Lønskov et al. describe a child homozygous for a deleterious variant in PARK7/DJ-1 presenting with RSV infection, brain inflammation, and seizures and explore the pathophysiology of PARK7 deficiency, underscoring the importance of PARK7 in human immune regulation and homeostasis.

## Introduction

Infection with respiratory syncytial virus (RSV) can cause bronchiolitis and pneumonia and is a major health problem worldwide (1, 2). While RSV infection is a relatively benign seasonal disease with flu-like symptoms in older children and adults, the remarkable virulence in infants and young children makes this virus responsible for a majority of childhood acute lower respiratory infection hospitalizations (3). Infection in infants has been associated with the development of asthma, wheezing, and other chronic lung diseases later in life (4). Risk factors predisposing to lower respiratory tract infection include preterm birth, low birth weight, chronic lung disease, congenital heart disease, inborn errors of metabolism, chromosomal abnormalities, and inborn errors of immunity (IEIs) (4). Involvement of the central nervous system (CNS) during RSV infection has been reported in some studies but is extremely rare (4).

RSV is an encapsulated, negative sense, single-stranded, non-segmented RNA virus related to metapneumoviruses in the family of Pneumovirinae with two major strains, RSV A and B, which circulate seasonally in the human population (4). The virus replicates in the epithelial lining of the nasopharynx before migrating to the lower respiratory tract, where it infects cells of the respiratory epithelium. Control of RSV replication is mediated by different cells of the innate immune system following the sensing of viral components like RSV RNA through toll-like receptors (TLRs), retinoic acid-inducible gene (RIG)-I, nucleotide-binding oligomerization domain-like receptors, and protein kinase R, which can induce an antiviral interferon (IFN) response among other control mechanisms (5). Cellular stress responses, including endoplasmic reticulum stress and the accumulation of reactive oxygen species (ROS), that are induced by the accumulation of misfolded proteins activating NF-κB–dependent inflammatory responses play an important role in the clearing of RSV (6). Programmed cell death/apoptosis can also contribute to RSV restriction by elimination of infected cells without excessive tissue damage (7), and RSV infection of human lung epithelial A549 cells was shown to induce apoptosis via the caspase pathway (8), the inducible nitric oxide synthase pathway (9), and the tumor necrosis factor-related apoptosis-inducing ligand pathway (10). However, RSV has developed immune evasion strategies to counteract these antiviral defense mechanisms, and several viral proteins have been shown to inhibit cell death (11). It is currently believed that much of the cell and tissue damage observed during RSV infection is caused by immunopathology mediated by RSV-induced alterations in inflammatory pathways and stress responses, together inducing a state of hyperinflammation (4).

A large and rapidly increasing number of IEIs predisposing to viral infections have been described (12, 13, 14, 15). For RNA viruses infecting the respiratory tract, such as influenza virus and severe acute respiratory syndrome coronavirus 2 (SARS-CoV-2), these include defects in type I IFN–inducing or responsive signaling pathways, such as TLR3, TLR7, TRAF3, IRF3, IRF7, IRF9, and IFNAR1/2 (15, 16, 17, 18, 19, 20, 21, 22), or phenocopies hereof in the form of neutralizing autoantibodies against type I IFN (23, 24, 25, 26). Likewise, defects in STAT2 may predispose to infection with influenza virus and SARS-CoV-2 (27, 28, 29). Moreover, homozygous deficiency of MDA5 (encoded by IFIH1) was identified in children with severe recurrent respiratory tract infections caused by rhinovirus, influenza virus, and RSV (30, 31). Collectively, these studies demonstrate a large but not incomplete overlap between IEI predisposing to infection by different respiratory viruses. However, beyond the classical type I IFN pathways, IEI in innate immunity conferring susceptibility to severe or complicated RSV infection have not yet been described. We therefore hypothesized that genetic defects involving alternative cellular and immune pathways may predispose to the severe and unusual course of viral infection in humans.

Here, we report a child with severe RSV infection complicated by inflammation and persistent high fever and development of acute encephalopathy with biphasic seizures and late reduced diffusion (AESD) homozygous for a novel loss-of-function variant in *PARK7* with a cellular phenotype revealing profound dysregulation of inflammation, stress pathways, cell death, and autophagy, possibly contributing to impaired antiviral control, hyperinflammation, and disease in the patient.

## Results

### A patient with severe RSV infection and CNS involvement

**Figure 1.**
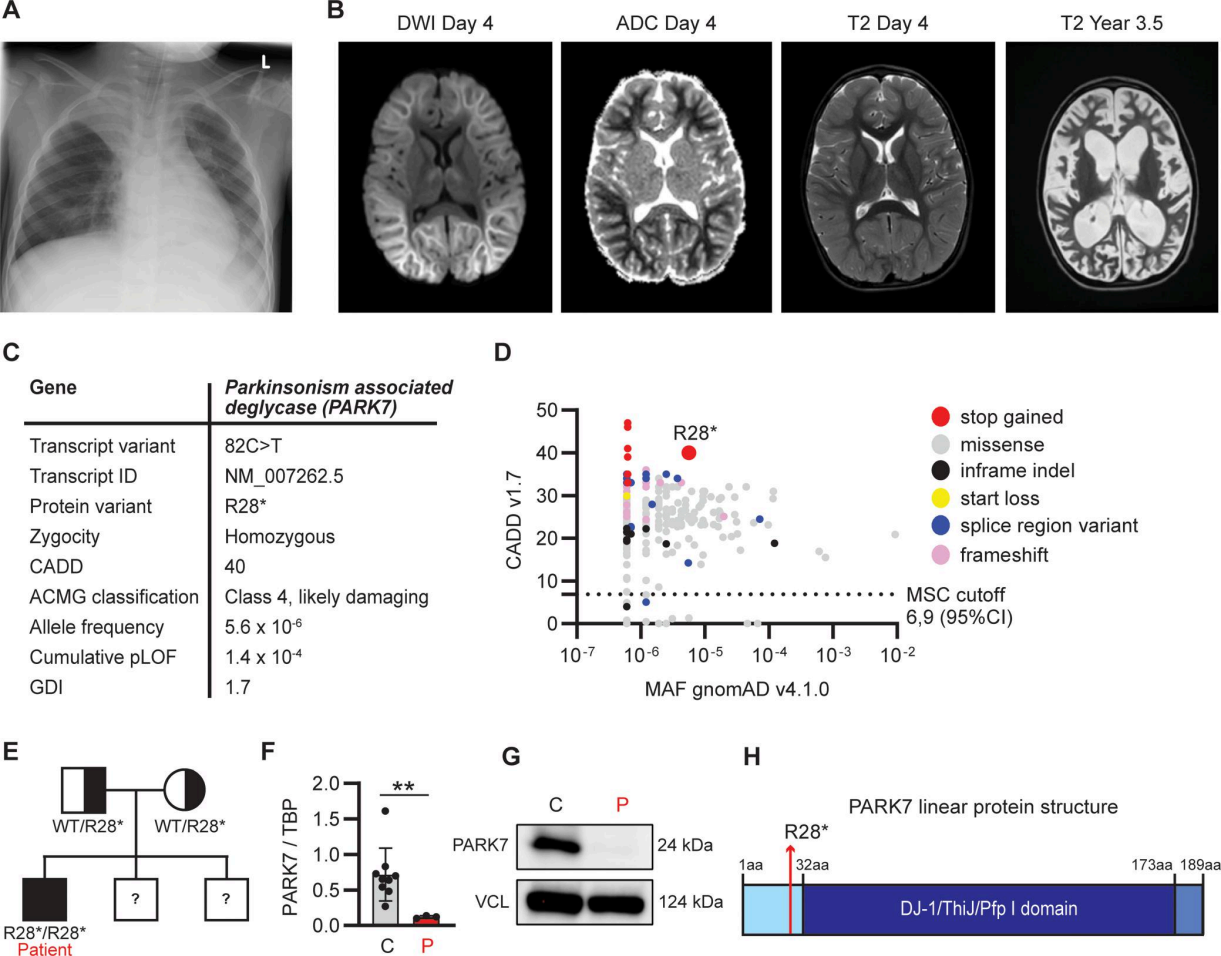
**Clinical and genetic findings in a 4-year-old patient with RSV-induced fever and seizures. (A)** Chest x-ray at day 1 of hospital admission showing pulmonary infiltration with fluid and atelectasis of the right upper lobe. **(B)** Magnetic resonance scan of the brain on day 4 after admission, showing extensive diffusion restriction predominantly involving subcortical white matter with sparing of the cortex described as “bright tree appearance.” **(C)** Genetic information of a novel rare variant in *PARK7* leading to a premature stop codon at R28, identified by WGS. ACMG, American College of Medical Genetics; GDI, gene damaging index. **(D)** PopViz results showing the CADD and MAF of pLOF variants in *PARK7* as identified in the patient (red) and variants reported in gnomAD, including variants leading to an early stop codon (stop gained, red), missense (grey), in-frame insertions and deletions (indel, black), start loss (yellow), alterations in mRNA splicing (splice region variant, blue), and frameshift (pink). The c.82C>T variant identified in the patient is indicated in the plot. No homozygous pLOF variants in PARK7 have been reported in gnomAD. The dashed line indicates the mutation cutoff score (MSC) at 95% confidence interval (CI). **(E)** Family pedigree for the PARK7 variant. The patient has two brothers whose DNA was not analyzed for the variant. **(F)** PARK7 RNA quantification relative to housekeeping gene (TBP) in patient PBMCs compared to healthy controls by RT-PCR. **(G)** PARK7 immunoblot of patient (P) fibroblast lysates compared to healthy control (C). Vinculin (VCL) was used as loading control. **(H)** Linear protein structure of PARK7. Amino acid (aa) residues and protein domains are indicated: the N-terminal region (light blue), DJ-1/ThiJ/Pfp I domain (dark blue), and the C-terminal region (blue). The red arrow indicates the location of PARK7 R28 that is mutated into a stop codon in the patient. DWI, diffusion-weighted imaging; ADC, apparent diffusion coefficient. Statistics were calculated using the unpaired two-tailed *T* test with Welch’s correction (E). **P < 0.01. Source data are available for this figure: SourceData F1.

A previously healthy 4-year-old boy (patient, P), born to Danish non-consanguineous parents, was admitted to hospital with high fever and long-lasting tonic-clonic seizures (see full medical history in the Materials and methods). Initially, c-reactive protein (CRP) was normal but rose over the disease course, accompanied by elevated leukocytes primarily due to lymphocytosis. Arterial puncture showed severe acidosis ascribed to respiratory insufficiency during long-lasting seizures, resolving within 2 h of admission. The patient was found to be reverse transcriptase PCR (RT-PCR) positive for RSV on a swab from the respiratory tract with no other viral pathogens identified. Chest x-ray at day 1 revealed pulmonary infiltration with fluid and atelectasis in the right upper lobe (Fig. 1 A). Saturation was around 96% on ventilatory support. Lumbar puncture early during the disease course (day 1) showed no abnormalities of the cerebrospinal fluid (CSF), and RT-PCR of the CSF was negative for viruses and bacteria. Magnetic resonance imaging (MRI) of the brain (day 2) was normal. Following an initial clinical improvement, the patient developed another tonic-clonic seizure and was hereafter increasingly encephalopathic. Due to persisting encephalopathy, an MRI of the brain was repeated at day 4, revealing cerebral edema with diffusion restriction (Fig. 1 B). Tracheal suctions from the patient remained positive for RSV RNA by RT-PCR for 35 days. Based on the clinical disease course and the imaging results, the patient was diagnosed with AESD. He was treated with high-dose corticosteroid and human immunoglobulin but only improved marginally. Day 6 after admission, the patient developed increased intracranial pressure, leading to the placement of an external ventricular drain (EVD); the disease course was later complicated by cerebral abscesses. After 3 years, the boy remains encephalopathic without any language, with cortical visual loss and severe epilepsy but has regained the ability to walk and eat.

### Identification of a biallelic variant in PARK7 and analysis of PARK7 protein expression

Due to the unusual course of the RSV infection with neurological symptoms, an IEI was suspected. The patient underwent a clinical immunological evaluation showing normal IgG, IgA, and IgM as well as normal numbers and fractions of T, B, and natural killer cells with normal T cell receptor excision circles (TRECs). Moreover, analyses showed a large fraction of activated T cells consistent with ongoing infection in the context of normal T cell proliferation following stimulation with anti-CD3 and anti-CD28. Whole-genome sequencing (WGS) revealed that the patient was homozygous for a variant in *Parkinsonism-associated deglycase* (*PARK7*, also termed *DJ-1*), c.82C>T p.Arg28* (NM_007262.5), causing a premature stop codon in the N-terminal part of the molecule, predicted to lead to nonsense-mediated mRNA decay, and classified as likely damaging (class 4) according to the American College of Medical Genetics guidelines (Fig. 1 C). The variant has not been reported in the literature; however, a submission is listed in the ClinVar database, in which the variant is classified as pathogenic. The variant is found in 9 out of 1,612,268 alleles in the gnomAD database. The variant has a high combined annotation dependent depletion (CADD) score of 40, well above the mutation significance cutoff of 6.9, suggesting a high degree of deleteriousness, and a low minor allele frequency (MAF) of 5.6 × 10^−6^, visualized by PopViz (Fig. 1 D) (32). In silico analysis demonstrates a low cumulative predicted loss of function (pLOF 1.4 × 10^−4^) and a low gene damaging index of 1.7, indicating that the accumulated mutational damage in *PARK7* in the healthy human population is low and the variant therefore predicted likely to be disease causing. gnomAD does not report any homozygous pLOF in PARK7. However, a homozygous deletion of the first five exons of the *PARK7* gene resulting in the absence of the protein was described in 2003 (33), and another homozygous frameshift variant (p.Ile31Aspfs*2) was reported in 2019 (34), although neither variant is currently listed in gnomAD. Seven homozygous missense variants are listed in gnomAD, of which three are predicted to be likely pathogenic by the AlphaMissense prediction score (p.Ser47Gly, p.Ala79Thr, and p.Arg156Gln) and one to be ambiguous (p.Ala104Ser). These four variants have each been reported once, indicating that homozygous pathogenic variants in *PARK7* are rare.

Trio analysis showed inheritance of the *PARK7* c.82C>T variant from each of the healthy variant-carrying parents (Fig. 1 E). WGS did not reveal pathogenic variants in any genes associated with IEI predisposing to viral infection, autoimmunity, or hyperinflammation (International Union of Immunological Societies criteria) (35). Quantification from patient peripheral blood mononuclear cells (PBMCs) demonstrated a significant decrease in PARK7 mRNA expression compared to in control PBMCs (Fig. 1 F), and immunoblotting of cell extracts from patient fibroblasts revealed complete absence of PARK7 expression (Fig. 1 G), with no smaller protein fragments detectable. PARK7 is a highly conserved protein of ∼20 kDa belonging to the DJ-1/ThiJ/Pfp I superfamily (Fig. 1 H) and is ubiquitously expressed in all cells but enriched in the brain, particularly in choroid plexus, tongue, and skeletal muscle (36). PARK7 is a multifunctional protein that acts as an antioxidant and oxidative stress sensor through several neuroprotective mechanisms (37).

### Hyperinflammatory cytokine and type I IFN responses in patient PBMCs

**Figure 2.**
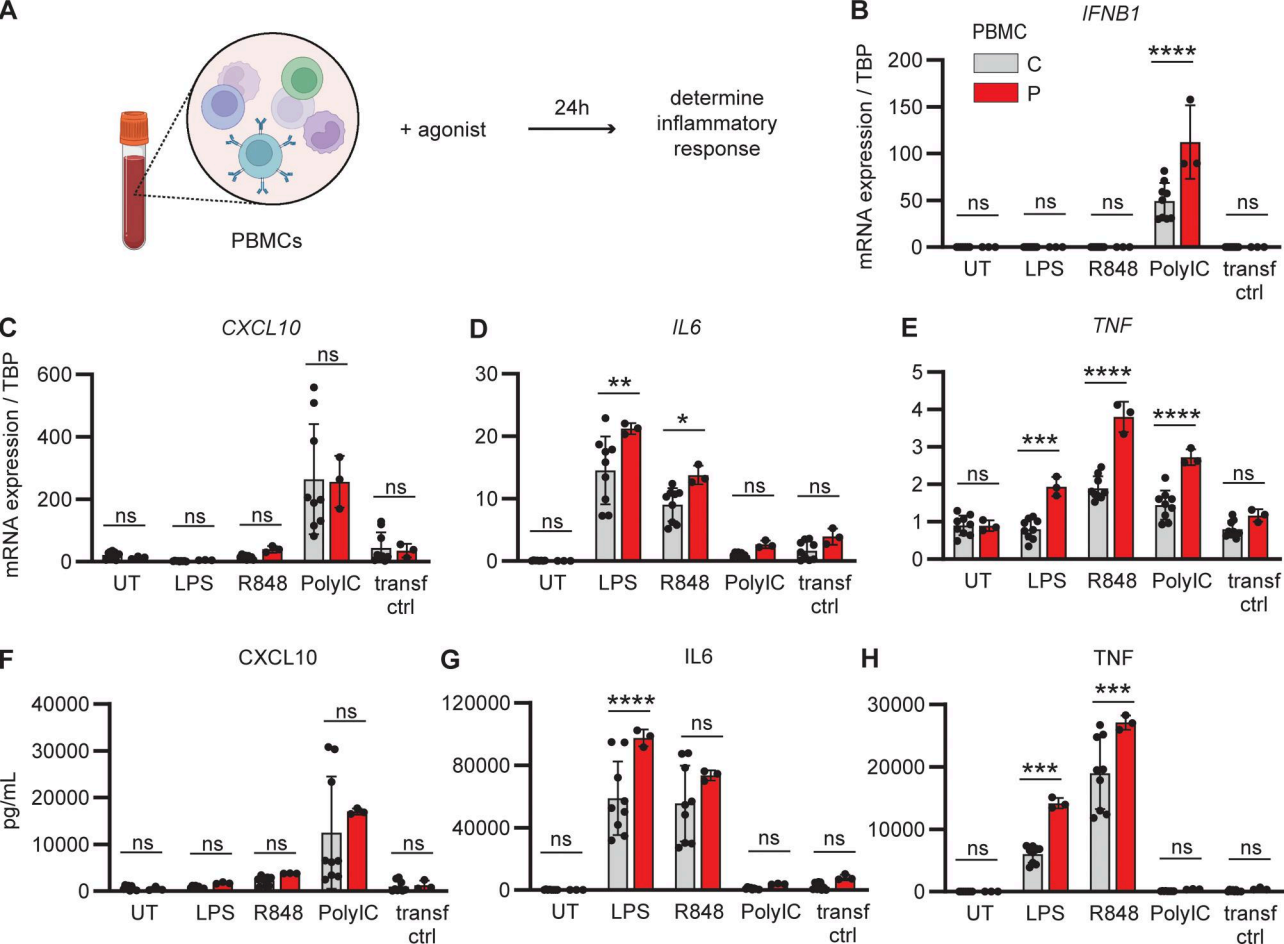
**Hyperinflammatory cytokine and IFN responses in patient PBMCs. (A–H)** PBMCs from the patient (red) and three controls (grey) were left untreated (UT), stimulated with TLR4 agonist LPS, TLR7/8 agonist R848, transfected with PolyIC for TLR3/MDA5/RIG-I stimulation, or mock transfected (transf ctrl). Cells were harvested 24 h after treatment, and RNA was isolated and analyzed for *IFNB1* (B), *CXCL10* (C), *IL6* (D), and *TNF* (E) gene transcription by RT-PCR, and cell culture supernatants were collected and analyzed for CXCL10 (F), IL6 (G), and TNF (H) protein by ELISA. Bars indicate mean values ± standard deviation (SD) of a single experiment performed in triplicates. Statistics were calculated using the ordinary One-way ANOVA with Šídák’s multiple comparisons test. * = P < 0.05, ** = P < 0.01, *** = P < 0.001, **** = P < 0.0001, and ns = not significant.

**Figure S1.**
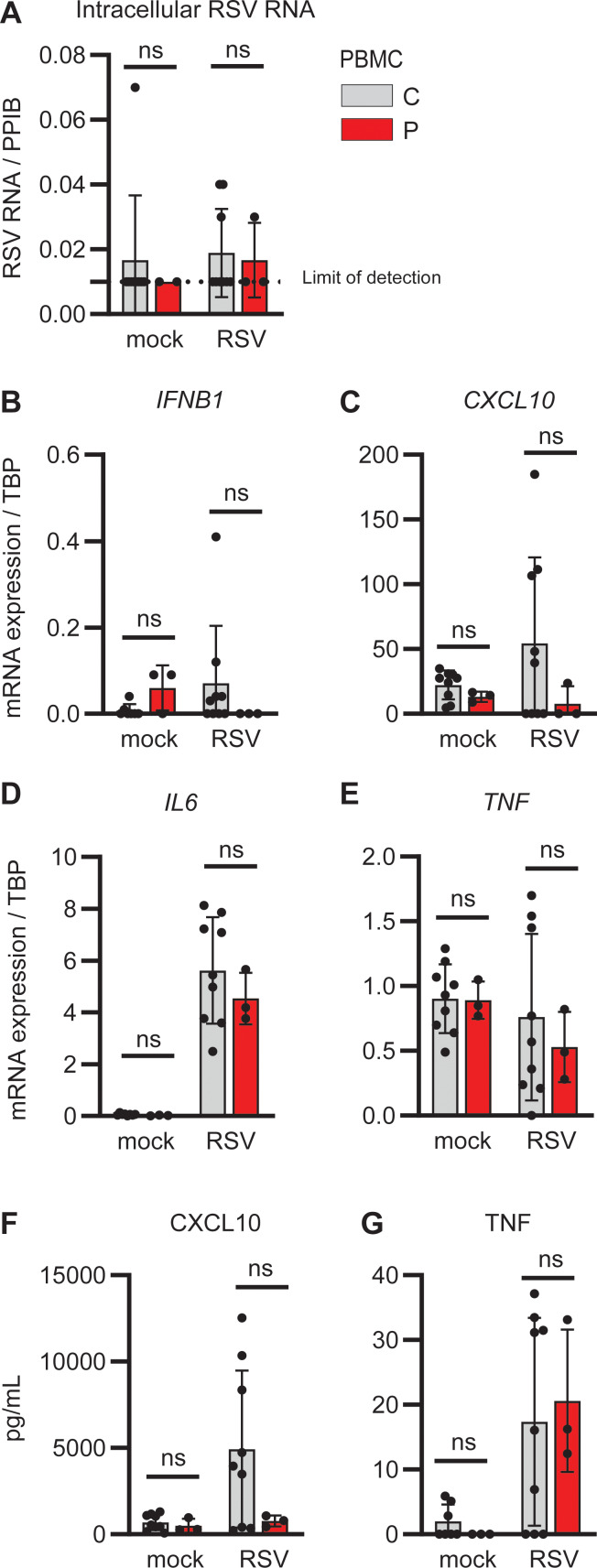
**Inflammatory cytokine and IFN responses in PBMCs following RSV infection.** PBMCs from the patient (P, red) and three controls (C, grey) were mock infected (mock) or infected with RSV (RSV-A strain Long, MOI 1). **(A–G)** Cells were harvested 24 h after treatment, and RNA was isolated and analyzed for intracellular RSV RNA (A), *IFNB1* (B), *CXCL10* (C), *IL6* (D), and *TNF* (E) gene expression by RT-PCR. Cell culture supernatants were harvested and analyzed for CXCL10 (F) and TNF (G) protein by ELISA. Bars indicate the mean ± SD values of a single experiment performed in triplicate. Statistics were calculated using the ordinary two-way ANOVA with Šídák’s multiple comparisons. ns = not significant. SD, standard deviation.

To first evaluate inflammatory cytokine responses, PBMCs from the patient (obtained ∼11 mo after hospital admission without any immunosuppressive or antiviral treatments) and three healthy controls were stimulated with the TLR4 ligand LPS, the TLR7/8 ligand, and R848 and by transfected PolyIC activating the cytosolic RNA sensors MDA5/RIG-I, representing innate RSV-sensing pathways. Cell transcripts were analyzed by RT-PCR after 24 h of stimulation, and expression of *IFNB1*, *CXCL10*, *IL6*, and *TNF* was quantified (Fig. 2 A). Compared to controls, patient PBMCs displayed significantly higher induction of *IFNB1* and *TNF* in response to stimulation with PolyIC, whereas *IL6* and *TNF* were enhanced in response to LPS and R848 (Fig. 2, B–E). Enhanced cytokine responses were confirmed by protein quantification in cell culture supernatants by ELISA, showing significantly enhanced IL6 after LPS stimulation and elevated TNF in response to both LPS and R848 (Fig. 1, F–H). Additionally, PBMCs (in which RSV does not cause productive infection) were challenged with RSV for 24 h (Fig. S1 A), which triggered only a weak inflammatory cytokine response that was similar between patient and control (Fig. S1, B–G). These results demonstrate that patient PBMCs display a broad hyperinflammatory response following stimulation with immunogenic pathogen-associated molecular pattern molecules (PAMPs) relevant for RSV infection.

### Reduced RSV-induced cell death in patient fibroblasts

**Figure 3.**
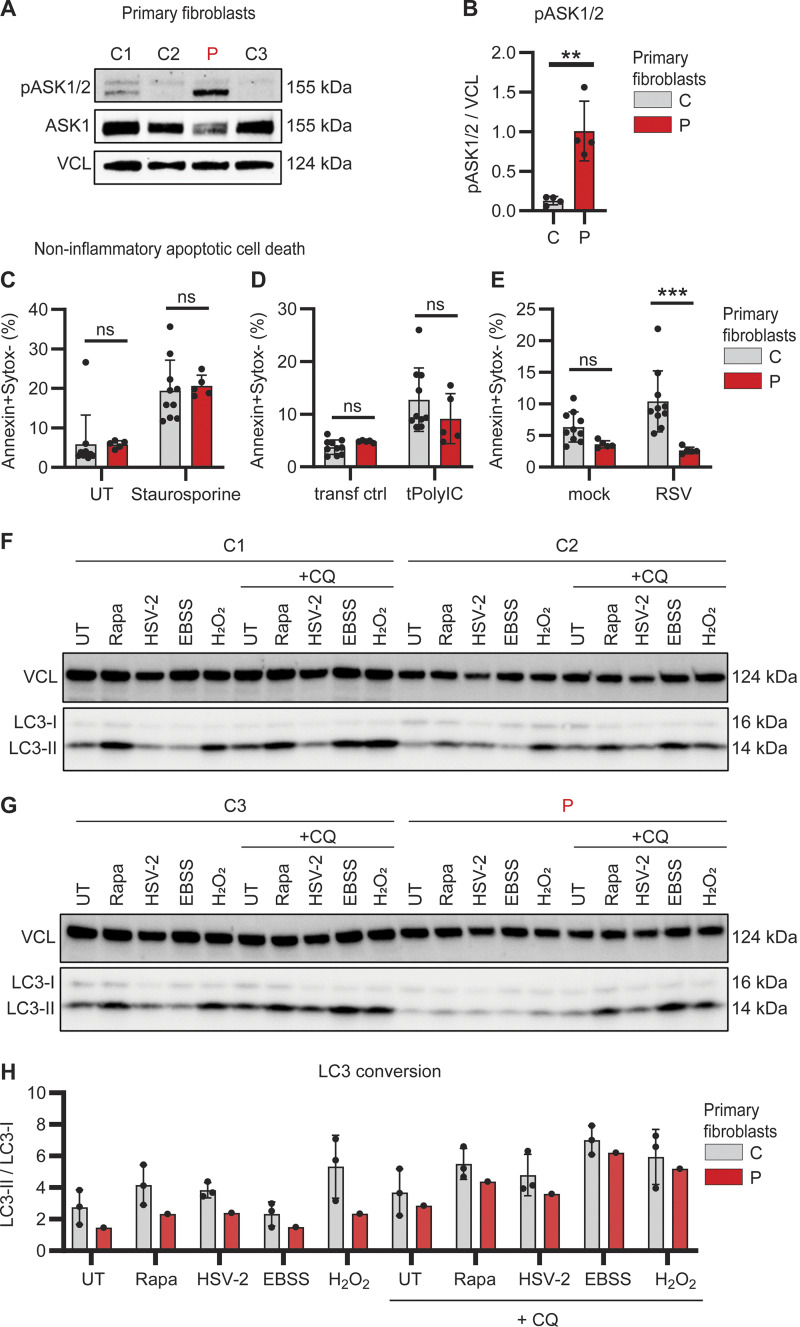
**Impaired RSV-induced apoptotic cell death and reduced autophagy in patient primary fibroblasts. (A and B)** Whole cell lysates from dermal fibroblasts from three controls (C, grey) and the patient (P, red) were analyzed for phosphorylation of ASK1/2 (pASK1/2), total ASK1, and vinculin (VCL) as loading control by immunoblotting (A). Graph depicting the densitometric quantification of pASK1/2 relative to total ASK1 (B). Bars show mean ± SD values of four experiments. **(C–E)** Primary fibroblasts from two controls (C, grey) and the patient (P, red) were stained with Annexin-V (apoptosis) and SYTOX Green (viability dye) to analyze induction of noninflammatory apoptotic cell death by flow cytometry. Cells were left untreated (UT) or treated with staurosporine (1 mM, 4 h) (C), mock transfected (transf ctrl) or transfected with PolyIC (tPolyIC, 500 ng/ml, 24 h) (D), or infected with mock virus (mock) or RSV (RSV-A strain Long, MOI 3, 24 h) (E). Bars show mean ± SD values of two experiments performed in duplicate and triplicate. **(F–H)** Primary fibroblast from three controls (C1–3) and the patient (P) were left untreated (UT), stimulated with rapamycin (Rapa, 500 nM, 24 h), HSV-2 (MS strain, MOI 1, 24 h), starved by culture in EBSS (4 h) or stimulated with H_2_O_2_ (300 nM, 30 min), in the absence or presence of chloroquine (+CQ, 20 μM, 4 h) to evaluate autophagy flux. Whole cell lysates were analyzed by immunoblot for LC3 lipidation and conversion of LC3-I into the smaller LC3-II with VCL as loading control. **(H)** Graph showing the ratio LC3-II/LC3-I as indicator for autophagy induction in primary fibroblasts from three controls (C, grey bars) and the patient (P, red). The ratio of LCR3-II/LC3-I was quantified using densitometry analysis of immunoblots in F and G. Bars show mean ± SD values. Statistics were calculated using the unpaired *T* test (B) and ordinary two-way ANOVA with Šídák’s multiple comparisons (C–E). ** = P < 0.01, *** = P < 0.001, and ns = not significant. SD, standard deviation. Source data are available for this figure: SourceData F3.

Since the hyperinflammatory response of patient PBMCs appeared not to be restricted to a single specific innate signaling pathway tested, we next investigated expression and activation of apoptosis signal-regulating kinase (ASK)-1 (also termed MAP3K5), an inflammatory pathway previously linked to the antioxidant function of PARK7 (38, 39). ASK1 can be activated under conditions of cellular stress, including oxidative stress, ultraviolet light, and TNF stimulation, to form ASK1 homo- or heterodimers with ASK2 (MAP3K6), thereby facilitating inflammatory cytokine production and apoptosis, respectively (40). Of special interest in this context, activation of ASK1 can be directly inhibited by PARK7 (38). Due to limited availability of patient PBMCs, ASK1/2 phosphorylation was evaluated in primary patient fibroblasts. Compared to control fibroblasts, base-line phosphorylation of ASK1/2 was strikingly and significantly increased in patient fibroblasts, suggesting constitutively elevated stress-signaling pathways (Fig. 3, A and B).

**Figure S2.**
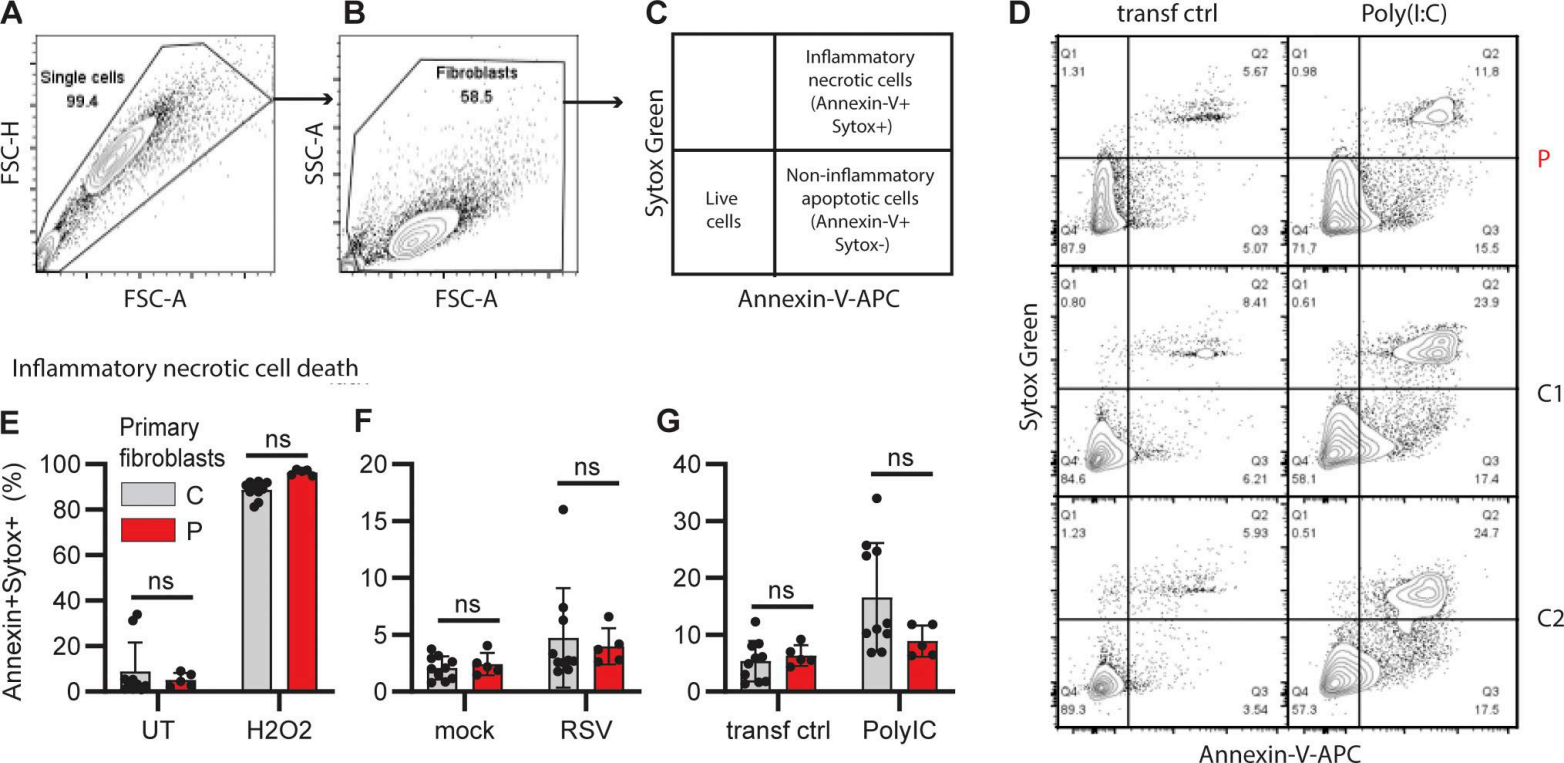
**Quantification of apoptotic and necrotic cells in patient and control fibroblasts. (A–D)** Representative density plots showing the analysis of noninflammatory apoptotic and inflammatory necrotic cells by flow cytometry. **(A and B)** Cells were gated for single cells by FSC-H and FSC-A (A), followed by cell selection to exclude cell debris from the analysis with SSC-A and FSC-A (B). These events were then analyzed for the frequency of apoptotic cells (Annexin-V positive, SYTOX Green negative, C) and necrotic cells (Annexin-V negative, SYTOX Green positive). **(D)** Representative density plots showing the frequency of Annexin-V– and SYTOX Green–positive cells in primary fibroblasts from the patient (P, top) and two controls (C1, middle and C2, bottom) that were mock transfected (transf ctrl, left) or transfected with PolyIC (500 ng/ml, 24 h, right). **(E–G)** Graphs showing the frequency of inflammatory necrotic cells in primary fibroblasts from two controls (C, grey) and the patient (P, red). Cells were left untreated (UT) or treated with H_2_O_2_ (2 mM, 24 h, E), infected with mock virus (mock) or RSV (RSV-A strain long, MOI 3, 24 h, F), or mock transfected (transf ctrl) or transfected with PolyIC (500 ng/ml, 24 h, G). Bars show the mean ± SD values of two experiments (E–G) performed in duplicate and triplicate. Statistics were calculated using the ordinary two-way ANOVA with Šídák’s multiple comparisons. ns = not significant. SD, standard deviation.

To follow up on this finding, we investigated cell death by staining for Annexin V and SYTOX Green by flow cytometry to quantify noninflammatory (apoptosis) and inflammatory cell death (necrosis), respectively (Fig. S2, A–D). Apoptosis was induced equally in both patient and control fibroblasts following treatment with the protein kinase C inhibitor staurosporine (Fig. 3 C) and transfection with PolyIC (Fig. 3 D). However, RSV-induced apoptosis was significantly reduced in patient fibroblast (Fig. 3 E). In contrast, inflammatory necrotic cell death in response to hydrogen peroxide (H_2_O_2_) treatment, PolyIC transfection, or RSV infection was similar in patient and control fibroblasts (Fig. S2, E–G). These results show that apoptotic noninflammatory cell death is impaired in the absence of PARK7, potentially causing insufficient clearance of virus-infected dead cells that may trigger secondary hyperinflammation.

### Dysregulated constitutive and inducible autophagy in patient fibroblasts

PARK7 has been attributed to having an important role in regulating autophagy and cellular homeostasis (41). To examine autophagic responses, primary control and patient fibroblasts were challenged with the autophagy inducers rapamycin, Earle’s balanced salt solution (EBSS)–induced starvation, H_2_O_2_, or herpes simplex virus (HSV)-2 infection, in the absence and presence of chloroquine to investigate autophagy and autophagy flux, respectively. Cells were collected, and autophagy induction was evaluated by LC3 lipidation and conversion of LC3-I into the smaller LC3-II as visualized by immunoblot and quantified to calculate the ratio of LC3-II/LC3-I. These data revealed reduction in constitutive and inducible autophagy in patient fibroblasts, as compared to control fibroblasts (Fig. 3, F–H). Given the pro-homeostatic and anti-inflammatory role of autophagy (42), this reduction in autophagy flux, although modest in vitro, may contribute to inflammation and immune dysregulation in PARK7 deficiency. Taken together, the results suggest that loss of PARK7 leads to elevated stress-kinase signaling combined with reduced apoptotic programmed cell death and autophagy, which might potentially contribute to enhanced hyperinflammation in the patient.

### Unaltered RSV replication in PARK7-deficient pulmonary A549 cells

**Figure S3.**
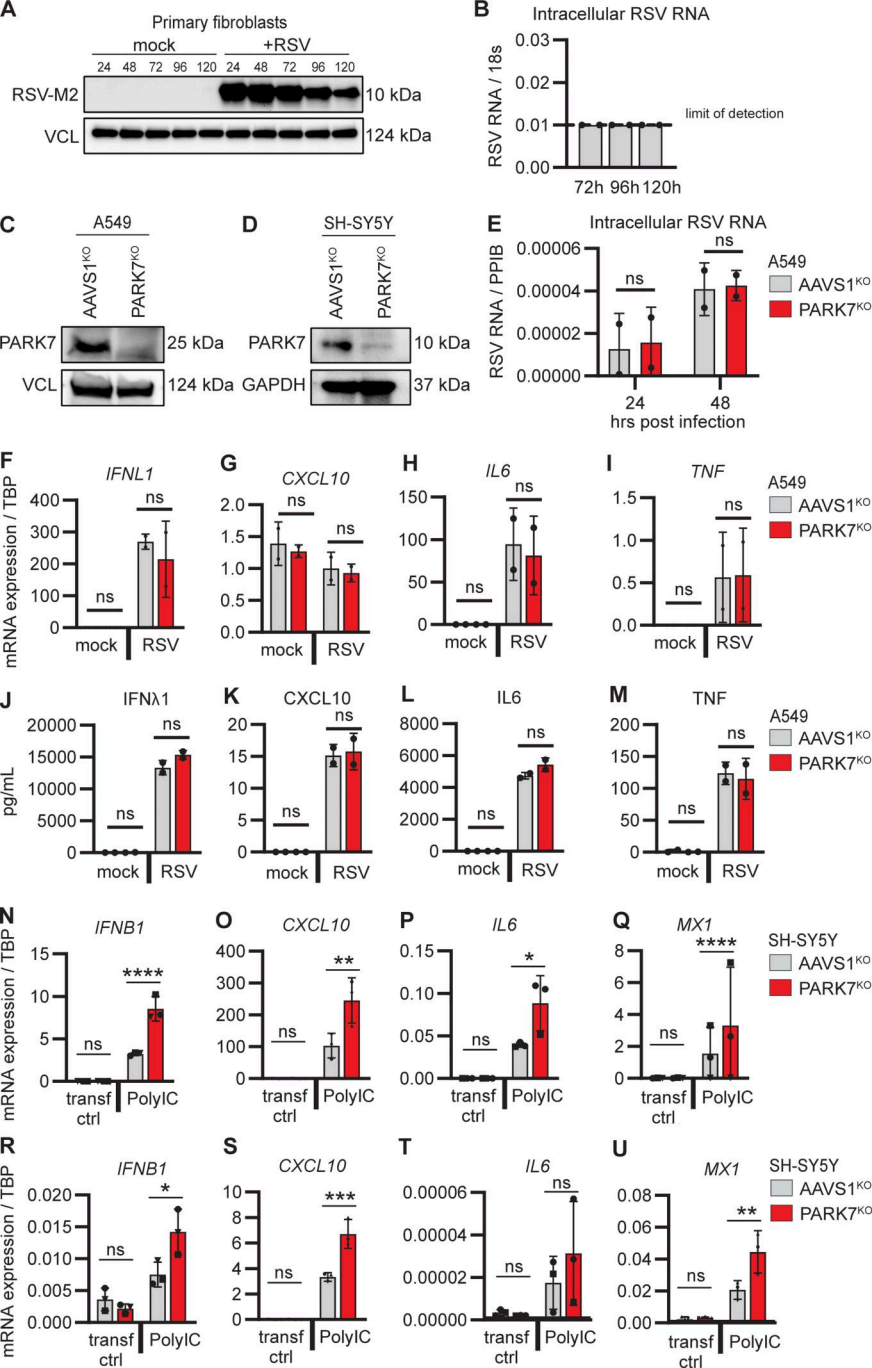
**Generation of PARK7-deficient A549 and SH-SY5Y cells and cytokine responses to RSV infection. (A and B)** Immunoblot and RT-PCR demonstrating absence of RSV replication in primary fibroblasts. Primary fibroblasts from a healthy donor were infected with RSV (RSV-A strain Long, MOI 1), and cells were collected at indicated time points. Whole cell lysates were analyzed by immunoblotting for expression of RSV matrix protein (RSV-M2) and compared to vinculin (VCL) as loading control (A), and cells were collected for RNA extraction and quantification of RSV RNA by RT-PCR (B). **(C and D)** Immunoblot demonstrating KO of PARK7 in A549 pulmonary cells (C) and PARK7 KO in neuronal SH-SY5Y cells (D). Vinculin (VCL, C) and GAPDH (D) were used as loading control. **(E)** Repeat of experiment shown in Fig. 4 A, showing additional replicates of two experiments performed in duplicate of human pulmonary A594 cells deficient in PARK7 (red) and control AAVS1^KO^ (grey) mock treated or infected with RSV (RSV-A strain Long, MOI 1) for 24 and 48 h, and cells were collected for RNA isolation and analysis of RSV RNA by RT-PCR. **(F–M)** PARK7^KO^ (red) and control AAVS1^KO^ (grey) A549 cells were mock treated or infected with RSV (RSV-A, strain Long, MOI 1) for 24 h. **(F–I)** Cells were collected for RNA isolation, and quantification of *IFNL1* (F), *CXCL10* (G), *IL6* (H), and *TNF* (I) gene transcription was performed by RT-PCR. **(J–M)** Cell culture supernatants were collected for IFNλ1 (J), CXLC10 (K), IL6 (L), and TNF (M) protein quantification by ELISA. **(N–U)** Repeats of experiment shown in Fig. 4, D–G, showing an additional two repeat experiments performed in triplicate (N–Q shows the second experiment and R–U shows the third experiment) where neuronal SH-SY5Y cells deficient in PARK7 or control AAVS1^KO^ were mock transfected (transf ctrl) or transfected with PolyIC (500 ng/ml). Cells were collected after 6 h, and RNA was isolated, and *IFNB1* (N and R), *CXCL10* (O and S), *IL6* (P and T), and *MX1* (Q and U) gene transcription quantification by RT-qPCR. Bars indicate mean ± SD values. Statistics were calculated using the two-way ANOVA with multiple comparisons. * = P < 0.05, ** = P < 0.01, *** = P < 0.001, and ns = not significant. SD, standard deviation. Source data are available for this figure: SourceData SF3.

**Figure 4.**
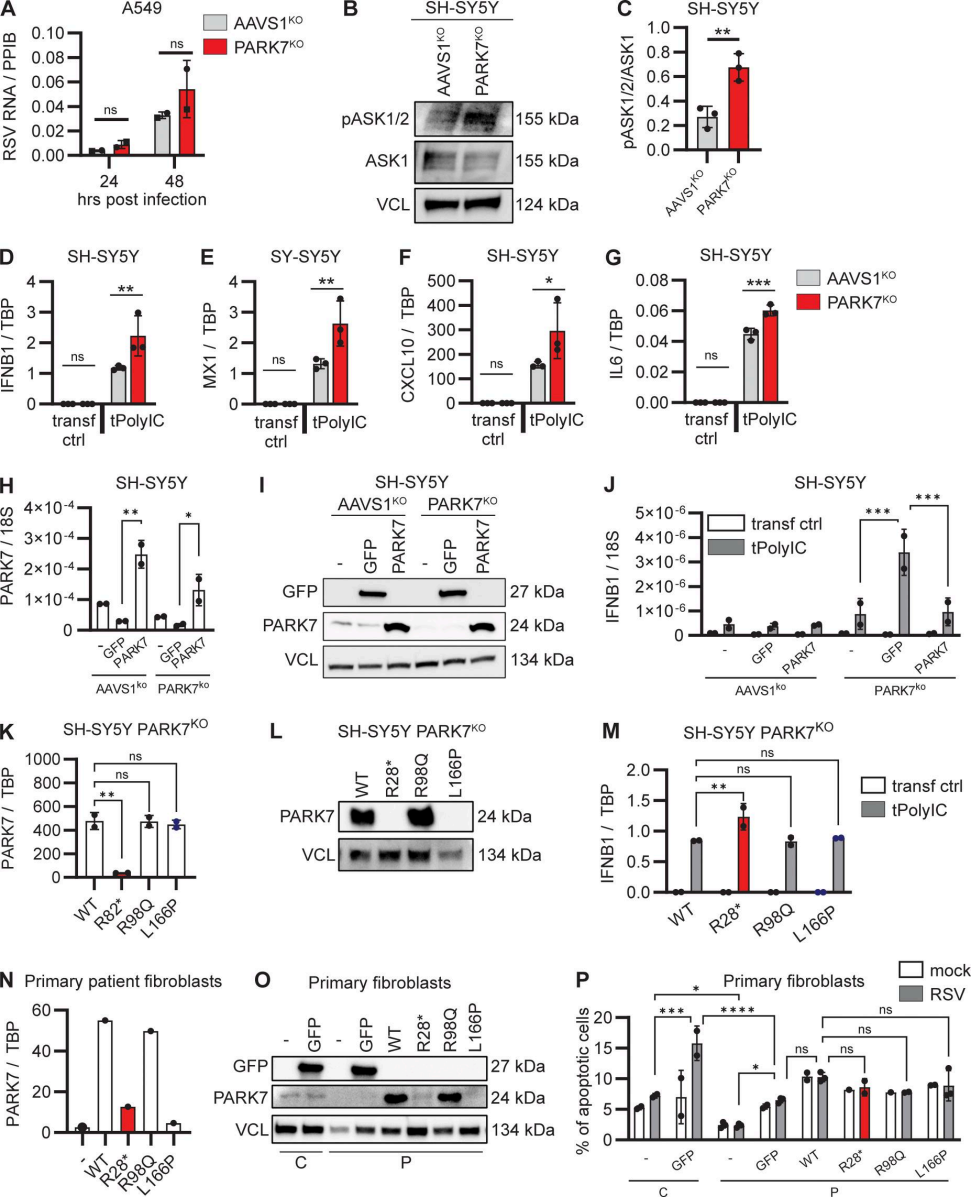
**Enhanced cellular stress pathways and hyperinflammatory cytokine responses in PARK7-deficient neuronal SH-SY5Y cells. (A)** Human pulmonary A594 cells deficient in PARK7 (red) and control AAVS1^KO^ (grey) were mock treated or infected with RSV (RSV-A strain Long, MOI 1) for 24 and 48 h, and cells were collected for RNA isolation and analysis of RSV RNA by RT-PCR. Bars indicate mean ± SD values of two experiments, performed in duplicate. Repeats of the experiments are shown in Fig. S3 E. **(B and C)** Whole cell lysates from SH-SY5Y with PARK7^KO^ (red) or AAVS1^KO^ (control, grey) were analyzed for phosphorylation of ASK1/2 (pASK1/2) and expression of total ASK1 and vinculin (VCL) as loading control by immunoblotting (B). Graph depicting the densitometric quantification of pASK1/2 relative to total ASK1 (C). Bars indicate the mean ± SD values of three experiments. **(D–G)** Neuronal SH-SY5Y cells deficient in PARK7 or control AAVS1^KO^ were mock transfected (transf ctrl) or transfected with PolyIC (tPolyIC, 500 ng/ml). Cells were collected after 6 h, and RNA was isolated, and *IFNB1* (D), *MX1* (E), *CXCL10* (F), and *IL6* (G) gene transcription quantification by RT-PCR. Bars indicate mean ± SD values of a representative experiment performed in triplicate. Repeats of the experiment are shown in Fig. S3, N–Q and R–U. **(H–J)** Neuronal SH-SY5Y cells deficient in PARK7 or AAVS^KO^ (control) were left untransduced (−) or were transduced with lentiviral vectors to express PARK7 or GFP (control). Cells were collected after 48 h for RNA isolation to determine PARK7 mRNA expression (H), and whole cell lysates were analyzed by immunoblotting for GFP and PARK7 expression with VCL as loading control (I). **(J)** Cells were transfected with PolyIC (tPolyIC, 500 ng/ml) and collected after 6 h for quantification of *IFNB1* transcription by RT-PCR. Bars indicate mean ± SD values of a representative experiment performed in duplicate. **(K–M)** Neuronal SH-SY5Y cells deficient for PARK7 were transduced with lentiviral vectors to express PARK7 WT, the patient PARK7 variant R28*, the R98Q variant frequent in the healthy population (MAF>10^−3^), and the L166P variant associated with Parkinson’s disease. Cells were collected for RNA isolation to determine PARK7 mRNA expression (K), and whole cell lysates were analyzed by immunoblotting for PARK7 expression with VCL as loading control (L). **(M)** Cells were transfected with PolyIC (tPolyIC, 500 ng/ml) and collected after 6 h for quantification of *IFNB1* transcription by RT-PCR. Bars indicate mean ± SD values of a representative experiment performed in duplicate. **(N–P)** Primary fibroblasts from the patient (P) were left untransduced (−) or were transduced with lentiviral vectors to express GFP, PARK7WT, PARK7 R28*, PARK7 R98Q, or PARK7 L166P. As control (C), primary fibroblasts from a healthy donor were left untransduced or were transduced to express GFP. Cells were collected for RNA isolation to determine PARK7 mRNA expression (N), and whole cell lysates were analyzed by immunoblotting for GFP and PARK7 expression with VCL as loading control (O). Cells were infected with mock virus (mock) or RSV (RSV-A strain Long, MOI 3, 24 h) and stained with Annexin-V (apoptosis) and 7-AAD (viability dye) to analyze induction of noninflammatory apoptotic cell death by flow cytometry (P). Bars show mean ± SD values of a single experiment performed in duplicate and triplicate. Statistics were calculated using the multiple paired *t* test (A), the unpaired *t* test (C), the one-way ANOVA (H and K), and ordinary two-way ANOVA with Šídák’s multiple comparisons (J, M, and P). * = P < 0.05, ** = P < 0.01, *** = P < 0.001, and ns = not significant. SD, standard deviation. Source data are available for this figure: SourceData F4.

Given our finding that RSV was unable to establish productive infection in PBMCs (Fig. S1 A) and primary human skin fibroblasts (Fig. S3, A and B), we genetically edited human pulmonary A549 cells with CRISPR/Cas9 to knock out (KO) *PARK7* and determined the effect of PARK7 loss upon RSV replication (Fig. S3 C). Pulmonary A549 cells with PARK7^KO^ or AAVS1^KO^, used as control for gene editing, were infected with RSV, and cells were collected 24 and 48 h after infection for quantification of intracellular genomic RSV RNA using RT-PCR. Intracellular RSV RNA increased from the 24 to the 48 h time point for both the control and the PARK7^KO^ cells, and there was no difference between control and PARK7-deficient cells at either time point (Fig. 4 A and Fig. S3 E). In addition, we evaluated the inflammatory cytokine profile but observed no differences between the PARK7^KO^ and control A549 cells (Fig. S3, F–M), nor could we detect ASK1 by immunoblotting (data not shown), which may explain why there was no hyperinflammatory response in this cell model. In summary, these findings do not suggest a direct lack of antiviral immunity in PARK7-deficient pulmonary A549 cells, instead indicating that pathology may be due to hyperinflammation.

### Hyperinflammatory responses in PARK7-deficient neuronal SH-SY5Y cells

To study viral control and inflammation upon RSV infection in CNS neurons, we used the neuronal cell line SH-SY5Y. PARK7^KO^ SH-SY5Y cells were generated using CRISPR/Cas9, and the PARK7 defect was confirmed by immunoblotting (Fig. S3 D). Additionally, we evaluated baseline ASK1/2 phosphorylation by immunoblotting and found that compared to control SH-SY5Y, phosphorylation of ASK1/2 in the untreated state was increased in PARK7-deficient SH-SY5Y (Fig. 4, B and C), recapitulating the results from PARK7-deficient patient fibroblasts. Both PARK7^KO^ and control cells were transfected with the double stranded RNA (dsRNA) mimic PolyIC to activate nucleic acid sensors, and cells were collected after 6 h for quantification of gene expression by RT-PCR. Compared to control cells, gene expression of *IFNB1*, *MX1*, *CXCL10*, and *IL6* was significantly increased in neuronal SH-SY5Y cells deficient for PARK7, recapitulating results from PARK7-deficient patient PBMCs, suggesting that loss of PARK7 contributes to hyperinflammation in this cell type and potentially in the brain (Fig. 4, D–G and Fig. S3, N–U).

### Reconstitution of PARK7-deficient cells reduces the hyperinflammatory phenotype

To explore whether the hyperinflammatory phenotype in patient cells and cell models could be caused by PARK7 deficiency, SH-SY5Y PARK7^KO^ were transduced with lentiviral vectors to express PARK7 or GFP, the latter as control for the transduction procedure. PARK7 expression was confirmed at the mRNA and protein level (Fig. 4, H and I), and cells were stimulated with PolyIC and analyzed for gene expression by RT-PCR. Compared to GFP-transduced control cultures, cells with reconstituted PARK7 WT showed a significant decrease in the polyIC-induced *IFNB1* response, the phenotype previously revealed in patient PBMCs and cell lines deficient in PARK7 (Fig. 4 J). These data demonstrate that reintroduction of PARK7 decreases the inflammatory responses in this neuronal cell model, indicating that PARK7 deficiency underlies a hyperinflammatory cellular phenotype.

To evaluate the impact of the pathogenic patient variant on the inflammatory response, SH-SY5Y PARK7^KO^ cells were transduced with lentiviral vectors to express the patient variant PARK7-R28*, in parallel the cells were transduced to express the Parkinson’s disease–related pathogenic variant PARK7-L166P (c.497T>C), not present in gnomAD (33), and PARK7-R98Q that frequently prevails in the healthy population and is predicted to be benign (c.293G>A, p.R98Q, MAF 9.32 × 10^−3^, CADD 20.9). PARK7 expression was confirmed at the RNA and protein level for PARK7 WT and R98Q, whereas R28* could not be detected (Fig. 4, K and L), consistent with our previous observations in patient primary fibroblasts (see Fig. 1 G). Expression of the Parkinson’s disease variant L166P was confirmed at RNA level but undetectable at protein level, likely caused by protein instability and subsequent degradation (43, 44, 45, 46). Compared to PARK7 WT expressing cells, polyIC transfection induced a significant increase in *IFNB1* expression in cells expressing the patient variant R28*, indicating that expression of the early truncated PARK7 variant contributes to a hyperinflammatory state in these neuronal cells. As hypothesized, polyIC-induced *IFNB1* induction in cells expressing the R98Q variant from the healthy population did not differ from cells expressing WT PARK7. Surprisingly, cells expressing the Parkinson’s disease variant L166P did not differ from cells expressing WT PARK7, possibly suggesting that this variant may not contribute to Parkinson pathology by affecting IFN production and inflammation.

Finally, to assess whether the reduced RSV-induced apoptosis in patient fibroblasts could be rescued by PARK7 reconstitution, patient primary fibroblasts were transduced with lentiviral vectors to express GFP (control), PARK7 WT, the patient variant R28*, the benign R98Q variant from the healthy population, and the Parkinson’s disease variant L166P, with healthy control fibroblasts transduced to express GFP in parallel cultures. PARK7 expression was confirmed at the RNA and protein level for PARK7-WT and R98Q but could not be detected for the R28* and L166P PARK7 variants (Fig. 4, N and O). In the absence of lentiviral transduction, RSV-induced apoptosis was reduced in patient fibroblasts, as compared to control fibroblast, as expected based on the previous results (Fig. 4 P). Expression of GFP by lentiviral vectors enhanced apoptosis in both control fibroblasts and patient fibroblasts; however, the level of apoptosis remained significantly reduced in patient compared to control cells. Complementation of patient fibroblasts with PARK7 WT moderately increased apoptosis, as compared to GFP-expressing patient fibroblasts, albeit not statistically significant and not to the same level as GFP expressing control cells, implying that reconstitution of PARK7 WT could partially rescue the apoptotic phenotype. The other PARK7 R98Q and L166P variants did not alter the level of apoptosis significantly, possibly due to different cellular effects of different variants, although an effect of the relatively low frequency of apoptotic cells in the experimental model cannot be excluded.

**Figure 5.**
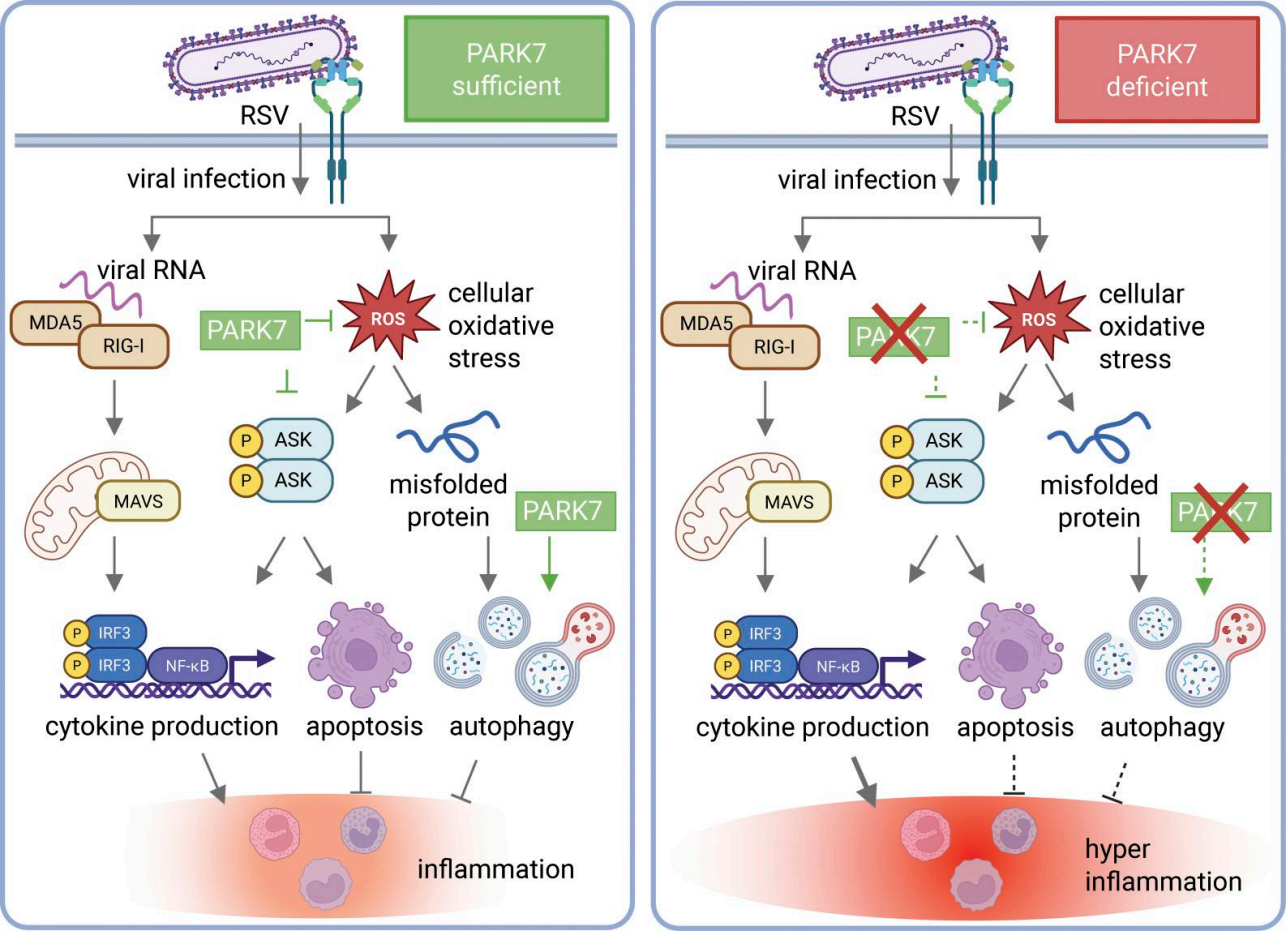
**Illustration of the working hypothesis on dysregulated responses to viral infection and damage by oxidative stress in PARK7 deficiency.** In healthy individuals with PARK7-sufficient cells (left), viral infection activates PPRs (MDA5/RIG-I) following sensing of viral RNA and oxidative stress signals (ROS) through stress kinases (ASK1), together inducing the production of inflammatory cytokines. Apoptosis and autophagy pathways reduce inflammation and facilitate a balanced cytokine and IFN responses to regain homeostasis. PARK7 (in green) can regulate ROS via Nrf-2 (47) and NADPH (48) (not shown) and inhibit ASK1/2 signaling pathways (49), thereby reducing inflammation. PARK7 can also modulate the activity of the specific autophagic receptor SQSTM1/p62 and contribute to the degradation of targeted proteins under oxidative stress conditions (41). In the patient with PARK7-deficient cells (right), the suppressive effect on ROS and the activating effect on autophagy are lost, resulting in enhanced inflammatory cytokine production (see Fig. 2 and Fig. 4) and reduced cellular apoptosis and autophagy (see Fig 3). Thus, PARK7 deficiency affects cellular responses during infection and cellular stress and is dominated by impaired apoptosis and autophagy, ultimately resulting in hyperinflammatory responses and pathology. NADPH, nicotinamide adenine dinucleotide phosphate; Nrf-2, nuclear factor erythroid 2-related factor 2.

Fig. 5 illustrates the working hypothesis for how PARK7 deficiency may cause dysregulated responses to viral infection and damage by oxidative stress. In healthy cells, viral infection elicits cytokine production via RLR activation alongside oxidative-stress mediated ASK-dependent apoptosis, which in turn serves to limit excessive inflammation. This balance appears to be disturbed in PARK7 deficiency, which is dominated by impaired apoptosis and autophagy, ultimately resulting in hyperinflammatory responses and pathology.

## Discussion

AESD is a rare clinical presentation in children with long-lasting seizures following a viral infection. Human herpes virus (HHV)-6/7 and influenza virus are the pathogens most frequently causing this condition (50), whereas AESD following RSV infection has been described in a few Asian children and in two cases reported in European children (50, 51, 52). A link between primary RSV infection, CNS inflammation, and neuronal damage was first observed in 1970, in a case study of 78 febrile children with and without seizures (53), and subsequently viral antibodies were detected in the CSF of patients with encephalitis (54, 55, 56, 57). Although AESD is extremely rare, other acute neurological symptoms, such as central apneas, seizures, and abnormalities in the CSF, are more frequent and amount to 2% in all RSV infection (58), increasing to 39% of RSV-positive patients on the pediatric intensive care unit in one study (4). These studies highlight the involvement of the CNS during severe RSV infection, although the virus cannot always be detected in the CSF, even in patients with severe neurological complications (58). While the number of patients reported with RSV-driven neurological pathologies remains low, most share commonalties of high levels of IL-6 in the CSF together with febrile seizures (59). In our study, RSV was not a strong inducer of inflammatory cytokine responses in PBMCs under the tested conditions (i.e., multiplicity of infection [MOI] 0.5 and 24 h of incubation). However, stimulation with PAMPs related to cell death and viral infection demonstrated clear hyperinflammatory cytokine responses, including IL6, in patient PBMCs.

It is currently unknown why patients in rare cases may experience neurological disease in relation to RSV infection. In addition, the precise mechanism whereby RSV might reach the CNS, as well as the neurotropism of the virus, has not yet been fully established. In mice, it was shown that viral load in the CNS decreases significantly after intranasal challenge with RSV, when the leukocyte integrin alpha subunit CD49d is blocked, indicating a potential “Trojan horse” mechanism by which RSV may invade the CNS via infected phagocytes (60). In the present study, patient cells did not show increased RSV replication, suggesting that the pathogenesis was dominated by oxidative stress and hyperinflammation, rather than loss of direct antiviral activity, which would be in agreement with the cerebral inflammation and edema, but absence of RSV detection in the CSF. In general, it is unresolved whether the severe brain damage seen in AESD is caused by fever, seizures, and hyperinflammation, or whether viral invasion and (undetectable) replication in the CNS is part of the pathogenesis. Indeed, some studies have suggested that an important aspect of the pathophysiological mechanism includes excitotoxic injury with delayed neuronal death (61, 62, 63, 64, 65).

Initially, PARK7 was described as a novel oncogene acting in cooperation with H-ras (66), and it was not until 2003 that loss of PARK7 was shown to be associated with severe neurodegeneration, which ultimately leads to early-onset Parkinson’s disease (33). PARK7-associated Parkinson’s disease is inherited in an autosomal recessive manner and accounts for ∼1% of all recessively inherited early-onset Parkinson’s disease cases (37). Most PARK7 variants causing early-onset Parkinson’s disease are missense mutations localized in the vicinity of highly conserved regions of the protein (37). The pathophysiological mechanism is believed to be due in part to the loss of the cytoprotective function against ROS exerted by PARK7 (67). In line with this, PARK7 is significantly upregulated in active astrocytes in multiple sclerosis patients, and PARK7 deficiency leads to loss of astrocyte anti-inflammatory functions (68, 69). Moreover, PARK7 is upregulated in epithelial cells infected with influenza virus H1N1, suggesting a role in antiviral immunity against this virus (70).

PARK7 has been implicated in multiple different biological functions. First, it has been established that PARK7 plays a role in the clearance of metabolites and damaged proteins, although no enzymatic function has been ascribed to PARK7 (71). Second, PARK7 has been implicated in the development of adaptive immunity, since loss of PARK7 in mice was reported to cause reduced amounts of CD4^+^ T cells and increased numbers of T regulatory cells (72). Third, PARK7 is involved in regulating several cell signaling cascades and can activate nuclear factor erythroid 2-related factor 2 (47), thereby reducing cellular oxidative stress (38), and PARK7 can inhibit ERK1/2 and ASK1/2 signaling pathways that mediate induction of IFNs and inflammatory cytokines in response to oxidative stress and viral infection (49), thereby in either case reducing inflammation. Fourth, PARK7 regulates nicotinamide adenine dinucleotide phosphate oxidase-dependent ROS production, which in turn can increase signaling via TLR4-mediated MAPK4 and NF-κB pathways to produce inflammatory cytokines (48). Together, these studies emphasize the diverse and complex roles of PARK7 in different cellular environments, with its protective role against ROS through modulation of cell death and anti-oxidative responses as one of the major functions (73).

In summary, we report findings from a child with a very unusual disease course manifesting as AESD following a severe RSV infection. WGS demonstrated that the child was homozygous for a rare loss-of-function variant in *PARK7*. Characterization of the cellular phenotype demonstrated major alterations and dysregulations in inflammatory stress responses, cell death, and autophagy pathways in patient cells, together likely conferring a pathological hyperinflammatory state. It is tempting to speculate that the RSV-induced fever and long-lasting seizures with hypoxia and acidosis might have triggered universal cell stress, contributing to the hyperinflammation, possibly aggravated by reduced homeostatic autophagy responses. This raises possibilities for prophylactic measures against severe RSV infection in susceptible individuals, either using the RSV vaccine, monoclonal antibodies, or passive transfer of immunity to newborns from mothers vaccinated during pregnancy (74, 75, 76). To our knowledge, this is the first association between severe RSV infection with hyperinflammation and CNS involvement and PARK7 deficiency. Based on these findings, we suggest that PARK7 deficiency may have contributed to the severe inflammatory CNS complication of RSV infection in this patient and propose that PARK7 deficiency, beyond the known link to Parkinson’s disease, may represent a novel IEI predisposing to hyperinflammation and pathology in response to viral infection in humans.

## Materials and methods

### Full medical history of the patient

A previously healthy 4-year-old boy (patient, P), born to Danish non-consanguineous parents, was admitted to hospital with high fever and long-lasting tonic-clonic seizures. Initially, CRP was normal but rose over the disease course to 30 mg/L (<8 mg/L), accompanied by elevated leukocytes 17.3 × 10^9^/L (4.5–12.5 × 10^9^L), primarily due to lymphocytosis (7.34 × 10^9^/L) (1.3–3.5 × 10^9^/L) but otherwise normal hematology, and normal liver and kidney function. Arterial puncture showed severe acidosis with pH 7.08 (7.35–7.45), pCO_2_ 10.3 kPa (5–7.1 kPa), and HCO_3_ 17.6 mmol/L (22.5–26.9 mmol/L), ascribed to respiratory insufficiency during long-lasting seizures, and resolved within few hours of admission. The patient was found to be RT-PCR positive for RSV of a swab from the respiratory tract. The tracheal suction sample was also tested for SARS-CoV-2, influenza A and B, rhinovirus, adenovirus, parainfluenza virus, enterovirus, and parechovirus, which were all negative. Chest x-ray at admission showed significant atelectasis and fluid corresponding to the right upper lobe, and new chest x-ray at day 3 revealed pulmonary infiltration with fluid in the left lower lobe. Saturation was around 96% on ventilatory support. Lumbar puncture early during the disease course (day 1) showed no abnormalities of the CSF, and RT-PCR of the CSF was negative for HSV1, HSV2, varicella zoster virus (VZV), human herpesvirus (HHV) 6/7/8, RSV, and enterovirus. MRI of the brain (day 2) was normal. Following an initial clinical improvement, the patient developed another tonic-clonic seizure on the second day and was hereafter increasingly encephalopathic. Due to persisting encephalopathy, MRI of the brain was repeated after another 2 days (day 4), which revealed cerebral edema with diffusion restriction (Fig. 1 A). Tracheal suctions from the patient remained positive for RSV RNA by RT-PCR at day 1, 7, 12, 17, 21, 32, and 35 and were finally negative at day 38. Based on the clinical disease course and the imaging results, the patient was diagnosed with AESD. He was treated with high-dose corticosteroid and human immunoglobulin but only improved marginally. 6 days after admission, the patient developed increased intracranial pressure, leading to the placement of an EVD. Later during the disease course, cerebral abscesses formed, likely due to the drain and prolonged treatment with prednisolone. After 3 years, the boy remains encephalopathic without any language, with cortical visual loss and severe epilepsy but has regained the ability to walk and eat.

There was no previous history suggesting increased susceptibility to infections. The boy had previously been hospitalized twice with febrile seizures at the ages of 1 and 3, respectively, possibly due to viral infections but without any further investigations at the time. Two older brothers in the family were healthy and currently have not been tested for the *PARK7* gene variant.

### Patient inclusion and material

The patient was included in the study by written parental consent alongside the parents’ inclusion in 2021. Whole blood samples were obtained in lithium heparin tubes for PBMC isolation by ficoll density gradient centrifugation using SepMate PBMC isolation tubes (#86460; STEMCELL Technologies) and frozen in liquid nitrogen. Control PBMCs were purified from healthy donors after obtaining written consent. A skin biopsy from the patient was taken under local anesthesia, and dermal fibroblasts were cultured at the Department of Clinical Genetics, Aarhus University Hospital (Aarhus, Denmark). Healthy control human dermal fibroblasts were obtained from PromoCell.

### WGS and bioinformatics

Genomic DNA from PBMCs from the patient and his parents was purified using the QIAamp DNA Blood Mini Kit (Qiagen) according to manufacturer’s instructions. WGS was performed using Illumina TruSeq PCR free library prep according to the procedures from the manufacturer (Illumina). The libraries were sequenced paired-end on a NovaSeq 6000 platform (Illumina) to a mean depth at no less than 30×. Alignment and variant calling were done using burrows-wheeler alignment (BWA) tool and genome analysis toolkit (GATK) with hg19 human genome as reference. VarSeq software (Golden Helix) was used for annotation and filtration of the genomic variants, and CNVkit, CNVnator, Manta, and Lumpy were used for copy number analysis. The data from the patients and his parents were filtered for variants in all known disease genes primarily according to the Online Mendelian Inheritance in Man database.

### Cell culture

SH-SY5Y (Research Resource Identification Portal [RRID]:CVCL_0019), primary dermal fibroblasts, A549-hACE2 (RRID:CVCL_A5KA), Hep-2 (RRID:CVCL_1906), and VeroE6/C1008 (RRID:CVCL_0574) were maintained as monolayer in Dulbecco’s modified Eagle’s medium (DMEM; Thermo Fisher Scientific). PBMCs were cultured in Roswell Park Memorial Institute (RPMI) 1640 medium (Thermo Fisher Scientific). DMEM and RPMI were supplemented with 10% heat-inactivated fetal calf serum, penicillin/streptomycin (p/s) (100 IU/ml; Sigma-Aldrich).

### Generation of polyclonal KO A549 and SH-SY5Y cells by CRISPR/Cas9 editing

PARK7 polyclonal KO cell lines were generated using the CRISPR/Cas9 gene-editing technology, as described previously (77). Briefly, ribonucleoprotein (RNP) complexes were generated by incubating 1.2 μg Cas9 protein (Integrated DNA Technologies) with 2 μg single guide RNA (sgRNA) (Synthego) at room temperature for 15–20 min. 100,000–200,000 cells were washed with PBS, resuspended in 20 μl of OPTI-MEM (Thermo Fisher Scientific), mixed with the RNP complexes, and transferred into a Nucleocuvette strip chamber (Lonza) and nucleofected using the Lonza 4D-Nucleofector System (program CA137 for SH-SY5Y and program CM138 for A549). The KO efficiency was determined by immunoblotting. The sequences of the synthetic guide RNA used for the KO were PARK7#1: 5′-GCA​TCT​TCA​AGG​CTG​GCA​TC-3′, PARK7#2: 5′-CAT​CAC​GGC​TAC​ACT​GTA​CT-3′, PARK7#3: 5′-GCT​CTG​GTC​ATC​CTG​GCT​AA-3′, PARK7#4: 5′-GAA​TGG​CTA​AAA​ATC​GAT​GT-3′, and AAVS1 (control sgRNA): 5′-GGG​GCC​ACT​AGG​GAC​AGG​AT-3′ (77).

### Infection and stimulation assays

PBMCs were seeded at 0.5 × 10^6^ cells per 96-U-shape well in 200 μl 10% RPMI+p/s and stimulated with 1 μg/ml LPS (InvivoGen) and 10 μg/ml R848 (InvivoGen), transfected with 2 μg/ml PolyIC low molecular weight (InvivoGen) using Lipofectamine 3000 (Thermo Fisher Scientific), and infected with RSV-A (MOI 0.5) or mock virus with the equivalent volume. Cells and cell culture supernatants were collected after 24 h of stimulation.

Fibroblasts were seeded at 0.5–1 × 10^5^ cells per 24-well in 500 μl 2% DMEM+p/s. For quantification of apoptotic and dead cells, cells were treated with 1 mM staurosporine (Sigma-Aldrich) and 2 mM H_2_O_2_ (Sigma-Aldrich), transfected with 500 ng/ml PolyIC high molecular weight (HMW, InvivoGen) using lipofectamine 3000, or infected with RSV-A (MOI 3) or mock virus of equivalent volume. Cells were collected after 24 h of stimulation, except for staurosporine-treated cells that were collected after 4 h, and apoptosis was determined by flow cytometry.

For quantification of autophagy induction, fibroblasts were treated with 500 nM rapamycin (Sigma-Aldrich), complete EBSS (Thermo Fisher Scientific), and 300 nM H_2_O_2_ or infected with HSV-2 (MOI 1) and ±20 μM chloroquine (Sigma-Aldrich). Cells were stimulated for 24 h, except for EBSS or chloroquine stimulation, which was 4 h, and whole cell lysates were collected for immunoblot analysis.

To determine if fibroblast can be productively infected, cells were seeded in 2% DMEM+p/s and infected the next day with RSV-A (MOI 1). Cells were collected 24–120 h after infection with 24-h intervals for analysis by either immunoblot or RT-PCR.

A549 PARK7^KO^ and AAVS1^KO^ cells were seeded at 4 × 10^5^ cells per 24-well in 50 μl 2% DMEM+p/s and infected the next day with RSV-A (MOI 1). Cells and cell culture supernatants were collected 24 and 48 h after infection for RNA isolation and ELISA, respectively.

SH-SY5Y PARK7^KO^ and AAVS1^KO^ cells were seeded at 1.4 × 10^5^ cells per 24-well in 500 μl 2% DMEM+p/s and transfected the next day with 500 ng/ml PolyIC HMW using Lipofectamine 3000. Cells were collected after 6 h for RNA isolation.

### Lentiviral expression of PARK7 in neuronal SH-SY5Y cells with PARK7 deficiency

The original PARK7 WT sequence was obtained and amplified from pGW1-Myc-DJ1-WT (RRID: Addgene_29347), subcloned into pccl-pgk lentiviral transfer vector via Gibson assembly, applying 25 ng of the pccl-pgk backbone and 50 ng of the PARK7 insert together with the NEBuilder HiFi DNA assembly master mix (New England Biolabs) according to the manufacturer’s instructions. PARK7 variants (c.82C>T, p.R28*; c.293G>A, p.R98Q; c497T>C, p.L166P) were purchased from GenScript Biotech and generated by side-directed mutagenesis in the PARK7-WT pGenLenti backbone (Clone ID: OHu26877C, NM_007262.5). Third-generation lentiviral vectors were produced as previously described (78). Briefly, 17 × 10^6^ HEK293T cells were seeded in 10% DMEM+p/s in a T175-bottle and transfected the following day with packaging plasmids (11.25 µg pMD2.G [Addgene_12259], 9 µg pRSV-Rev [RRID:Addgene_12253], and 39 µg pMDLg/pRRE [RRID:Addgene_12251]) and 39 µg pccl-pgk-PARK7 or pccl-pgk-GFP (78) lentiviral transfer vectors with a final concentration of 25 µg/ml polyethylenimine (PEI MAX, Kyfora Bio). 6 h after transfection, the medium was changed. Virus-containing supernatants were harvested 48 and 72 h after infection, filtered through 0.45-µm filters, and pooled for titration by quantification of the number of lentiviral integrations in HEK293T.

For reconstitution experiments, SH-SY5Y PARK7^KO^ and AAVS1^KO^ cells were seeded at a density of 1 × 10^5^ cells per 24-well in 2% DMEM+p/s. The next day, cells were transduced with lentivirus (10 MOI) with 8 µg/ml polybrene (Merck Millipore), and fresh medium was added after 24 h. Whole cell lysates were collected 48 h after transduction, and cells were transfected with 500 ng/ml PolyIC HMW using Lipofectamine 3000, which were collected after 6 h for RNA isolation. For reconstitution experiments with PARK7 variants, SH-SY5Y PARK7^KO^ and AAVS1^KO^ cells, and patient and control fibroblasts were seeded at 0.75 × 10^5^ cells/24-well in 2% DMEM+p/s. The next day, cells were transduced with lentivirus (10 MOI) with 8 µg/ml polybrene (Merck Millipore), and fresh medium was added after 24 h. Transduced cells were cultured for up to 3 wk and reseeded at 1 × 10^5^ cells/well for SH-SY5Y cells and 5 × 10^4^ cells/well for fibroblasts. SH-SH5Y cells were transfected with PolyIC as described above. Fibroblasts were infected with RSV-A (MOI 3) or mock virus of equivalent volume, collected after 24 h, and apoptosis was determined by flow cytometry.

### Virus

Virus propagation was conducted using Hep2 cells for RSV (RSV-A strain Long) and Vero cells for HSV-2 (MS strain), as described previously (79, 80). Briefly, cells were seeded in 2% DMEM+p/s and infected at a MOI of 0.05 (RSV) or 0.03 (HSV-2). A parallel flask was left uninfected to generate RSV mock virus, which followed the same procedure as infected cultures. At ∼50% cytopathic effect (CPE) for RSV and near 100% CPE for HSV-2, the cells were freeze-thawed, the supernatant was clarified of cellular debris by centrifugation and, for HSV-2, passed through a 0.45-μm filter, aliquoted, and stored at −80°C. The infectious virus titer was determined using a limiting dilution assay (RSV) or a plaque assay (HSV-2). RSV-A strain Long was kindly provided by Lara Schwab and Patrick Reading, University of Melbourne, Melbourne, Australia.

### Limiting dilution assay

The limiting dilution assay was used to determine the amount of infectious RSV in generated virus stocks. Briefly, 2 × 10^4^ Hep2 cells were seeded in 96-well flat-bottom plates in 2% DMEM+p/s. The next day, virus was added to the cells with a final dilution range of 10^−1^–10^−11^ in octuplicates. After 4 days, each well was evaluated for CPE using a standard light microscope, and the tissue culture infectious dose 50 (TCID50/ml) was calculated using the Reed and Muench method (81). The TCID50/ml values were multiplied by factor 0.7 to convert them into the mean number of plaque-forming units (PFUs)/ml (ATCC—Converting TCID50 to PFUs).

### Plaque assay

The plaque assay was used to determine the amount of infectious HSV-2 in generated virus stocks. Briefly, 8 × 10^5^ VeroE6 cells were seeded in 6-well flat-bottom plates in 2% DMEM+p/s. The next day, virus was added to the cells with a final dilution range of 10^−3^–10^−8^ in duplicates. 1 h after infection, Hizentra (CSL Behring) was added at a final concentration of 0.33 mg/ml. After 3 days, media was removed, and cells were stained using crystal violet stain (1:2:7 vol/vol/vol, crystal violet solution [Sigma-Aldrich], absolute ethanol, and deionized H_2_O); plaques were counted using a standard light microscope, and the PFU titer was calculated by the following formula: #plaques × dilution factor virus stock/volume virus added = PFU/ml.

### RNA isolation and RT-PCR

RNA was isolated using the High Pure RNA Isolation Kit (11828665001; Roche) or the NucleoSpin 96-well RNA extraction kit (Macherey-Nagel). RT-PCR was performed with the applied Biosystems TaqMan RNA to CT One Step Kit (4392938; Thermo Fisher Scientific) with primers: TBP(Hs00427620_m1), IFNB1(Hs01077958_s1), CXCL10(Hs01124251_g1), TNF(Hs00174128_m1), IL6(Hs00174131_m1), MX1(Hs00895608_m1), and IFNL1(Hs00601677_g1). Additionally, cDNA was synthesized using the iScript gDNA Clear cDNA Synthesis Kit (Bio-Rad), and the PCR was performed using the PowerUp SYBR Green Master Mix for quantitative PCR (Applied Biosystems) with primers: RSV(Fw#349132_N3070_[D12], Rev#349132_N3070_[E01]; Invitrogen), PPIB(FW#SS842083-06, Rev#SS842005-48; Biosearch), and 18 s(Hs03928985_g1). RNA was normalized to TATA-Box binding protein (TBP), peptidylprolyl Ieptidylprolyl isomerase B (PPIB), or 18s using the formula 2^(Ct[target]–Ct[control]).

### Western blotting

Whole cells were lysed in radioimmunoprecipitation assay lysis buffer (Thermo Fisher Scientific) supplemented with PhosSTOP (Merck), Complete Ultra protease inhibitor (Roche), Sodium fluoride (Avantor), and Benzonase Nuclease (Sigma‐Aldrich). Samples were diluted with Laemmli sample buffer (Sigma‐Aldrich), incubated at 95°C for 4 min, loaded on a 4–20% or 10% Criterion TGX Precast Midi Protein Gel in Nu PAGE MOPS SDS running buffer (Thermo Fisher Scientific), and transferred to a Trans‐Blot Turbo Midi polyvinylidene fluoride (PVDF) Transfer membrane (Bio‐Rad) using the Trans‐Blot Turbo Transfer System (Bio‐Rad). The following primary antibodies were used: anti-PARK7/DJ1 (#ab18257, RRID:AB_444361; Abcam), anti-RSV M2-1 (#ab94805, RRID:AB_10678103; Abcam), anti-LC3 (#2775, RRID:AB_915950; Cell Signaling Technology [CST]), anti-Vinculin (#V9131, RRID:AB_477629 or CST #13901, RRID:AB_2728768; Sigma-Aldrich), anti-GAPDH (#9485, RRID:AB_307275; Abcam), anti-ASK1 (#ab45178, RRID:AB_722915; Abcam), and anti-phosphorylated ASK1/2, kindly provided by H. Ichijo, The University of Tokyo, Tokyo, Japan, targeting Thr838 in human MAP3K5 and Thr806 in human MAP3K6 (82). As secondary antibodies, peroxidase‐conjugated donkey‐anti‐rabbit and donkey‐anti‐mouse were used (#711‐036‐152, RRID:AB_2340590, and #715‐036‐150, RRID:AB_2340773; Jackson Immuno Research). Specific bands were quantified by densitometry using the Image J software.

### Flow cytometry

Quantification of apoptotic and dead cells was performed using flow cytometry. Briefly, 5 × 10^4^ cells were washed twice with Annexin-V binding buffer (#422201; BioLegend) and stained for viability with 60 nM SYTOX Green (#S34860; Thermo Fisher Scientific) or 5 μl 7-AAD (#420403; BioLegend) and 2.5 μl Annexin-V-APC (#640941; BioLegend) or Annexin-V-PE (# 640908; BioLegend) in 50 μl Annexin-V binding buffer for 15 min in the dark, followed by one wash with Annexin-V binding buffer. Fluorescent intensity was measured with a NovoCyte 3000 flow cytometer equipped with three lasers (405, 488, and 640 nm) and 13 photomultiplier tubes (PMT) detectors (ACEA Biosciences, Inc.). Data were analyzed using FlowJo version 10.10.0 (BD Biosciences).

### Protein determination in supernatants by ELISA

Proteins were quantified using the Human DuoSet ELISAs for IL‐6, TNF, CXCL10, and IFNλ1 (R&D Systems), according to the manufacturer’s instructions on a Synergy HTX multi‐mode plate reader (BioTek) using the Gen5 version 3.04 program.

### Statistical analysis

The differences between experimental conditions of groups were analyzed using GraphPad Prism (Version 10) with the type of test indicated in each figure legend after outliers were defined. P values ≤0.05 were considered significant: * = P < 0.05; ** = P < 0.01; *** = P < 0.001.

### Online supplemental material

Fig. S1 shows the inflammatory cytokine and IFN responses in PBMCs following RSV infection. Fig. S2 shows the quantification of apoptotic and necrotic cells in patient and control fibroblasts. Fig. S3 shows the generation of PARK7-deficient A549 and SH-SY5Y cells and cytokine responses to RSV infection.

## Supplementary Material

SourceData F1 is the source file for Fig. 1.

SourceData F3 is the source file for Fig. 3.

SourceData F4 is the source file for Fig. 4.

SourceData FS3 is the source file for Fig. S3.

## Data Availability

To protect patient confidentiality in accordance with the European Union General Data Protection Regulation (GDPR) and Danish data protection laws, sequencing data and other information are available via controlled access according to national GDPR rules upon request to the corresponding author. All other data are available in the article itself and the accompanying supplementary materials.
