# Supplementary figures and images for: Viral infection and brain inflammation with seizures in PARK7 deficiency
Source: J Hum Immun. 2025 Dec 26;2(2):e20250044. doi: 10.70962/jhi.20250044 (PMC12889338; doi:10.70962/jhi.20250044)

1G

Fibroblast  
C P      samples not used in figure

PARK7

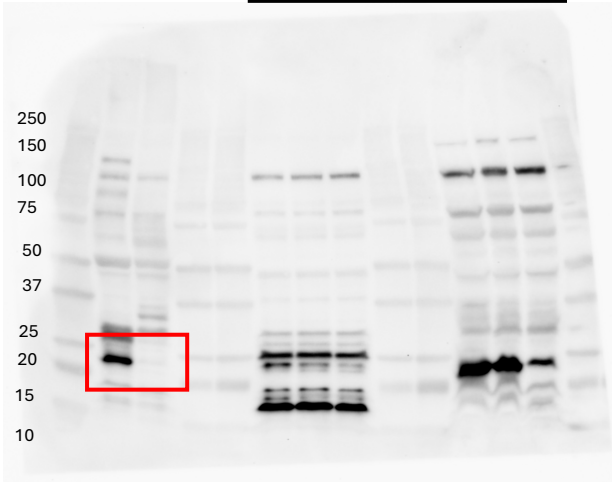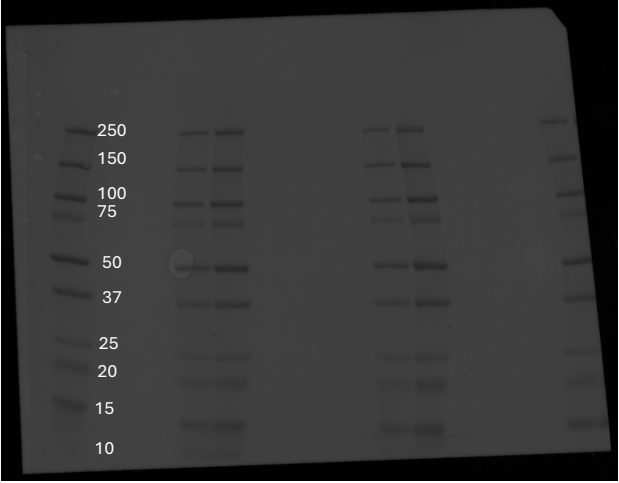

VCL

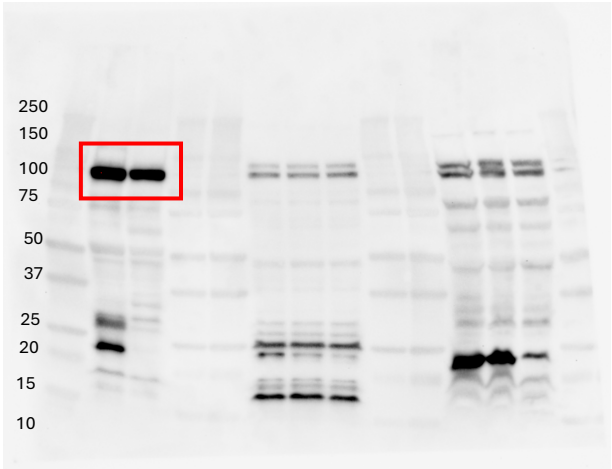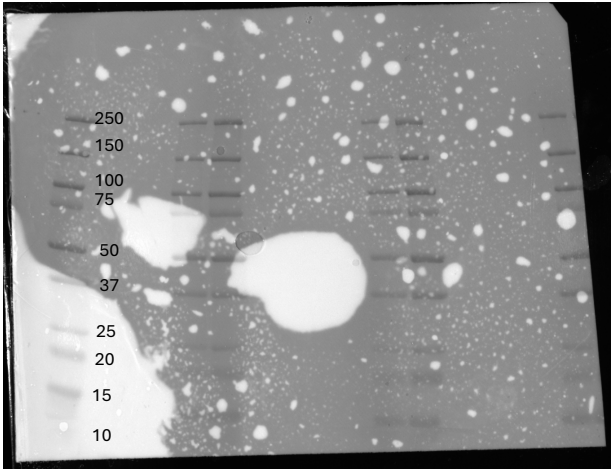

Supplement: SourceData F1 — is the source file for Fig. 1. [file jhi_20250044_sourcedataf1.pdf]

Primary fibroblasts

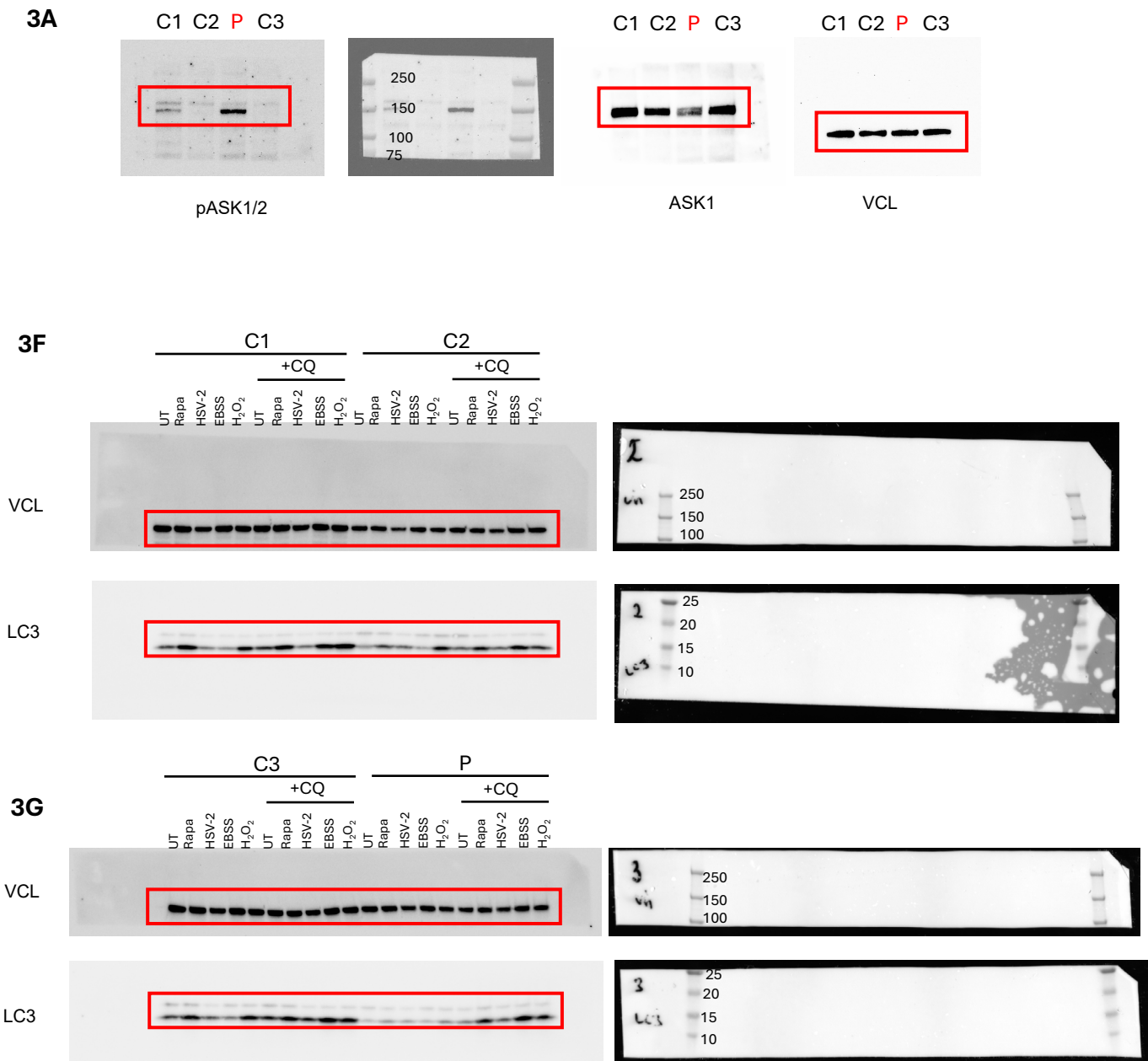

Supplement: SourceData F3 — is the source file for Fig. 3. [file jhi_20250044_sourcedataf3.pdf]

S3A

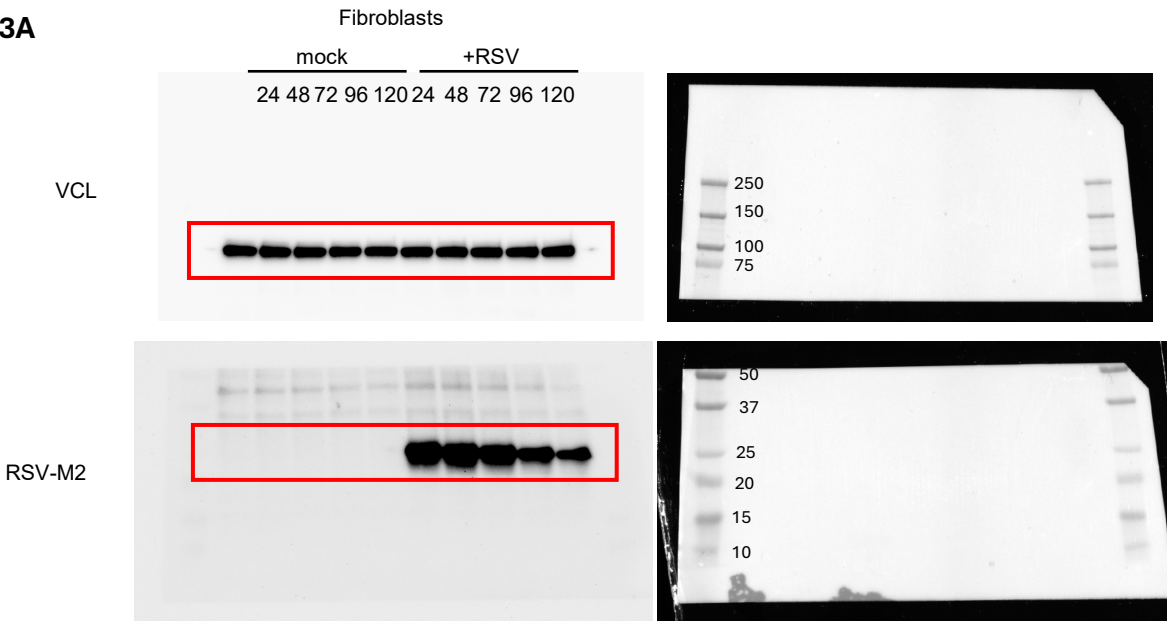

S3C

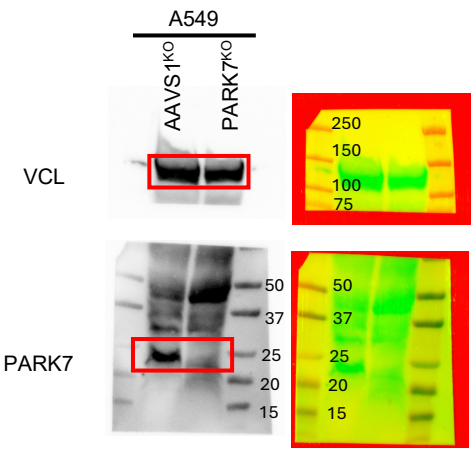

S3D

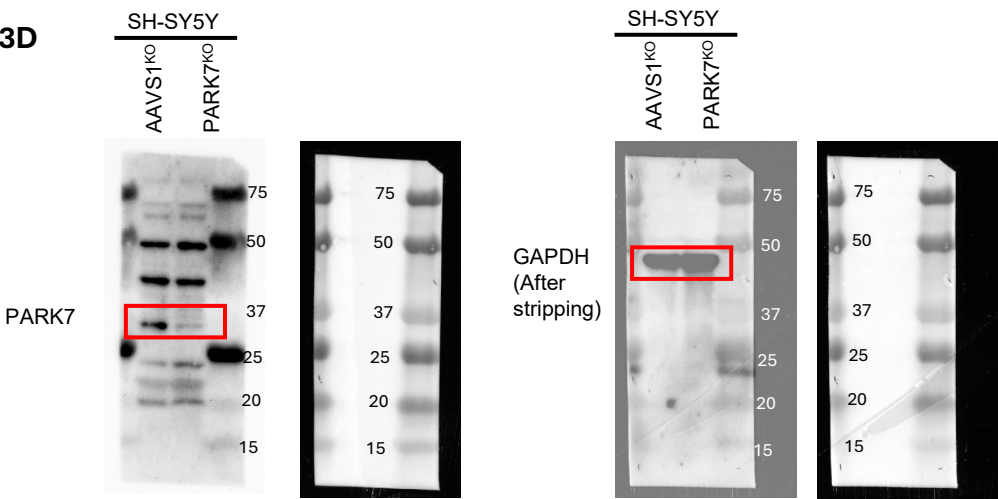

Supplement: SourceData FS3 — is the source file for Fig. S3. [file jhi_20250044_sourcedatafs3.pdf]
