# Supplementary material for: Viral infection and brain inflammation with seizures in PARK7 deficiency
Source: J Hum Immun. 2025 Dec 26;2(2):e20250044. doi: 10.70962/jhi.20250044 (PMC12889338; doi:10.70962/jhi.20250044)
Supplement: SourceData F4 — is the source file for Fig. 4. [file jhi_20250044_sourcedataf4.pdf]

## SourceDataF4

**4B**

SH-SY5Y

Other samples not used in figure

AAVS1$^{KO}$ PARK7$^{KO}$

pASK1/2

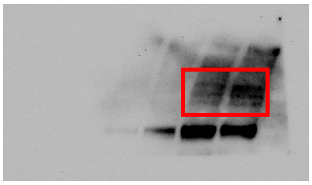

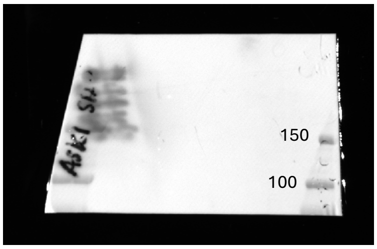


GAPDH

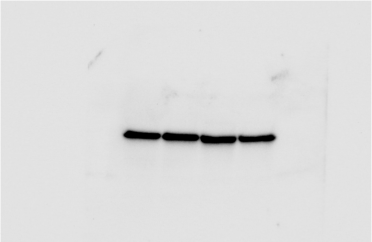

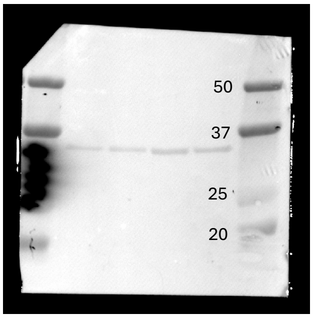


GAPDH not shown in figure

SH-SY5Y

Other samples not used in figure

AAVS1$^{KO}$ PARK7$^{KO}$

ASK1

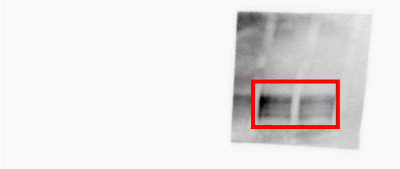

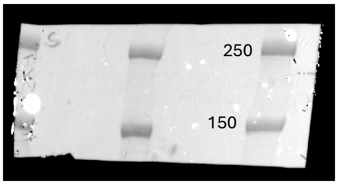


VCL

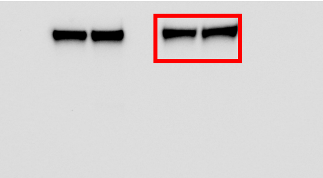

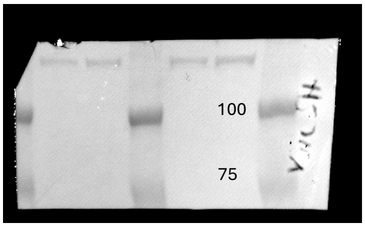

**SourceDataF4**

**4I**

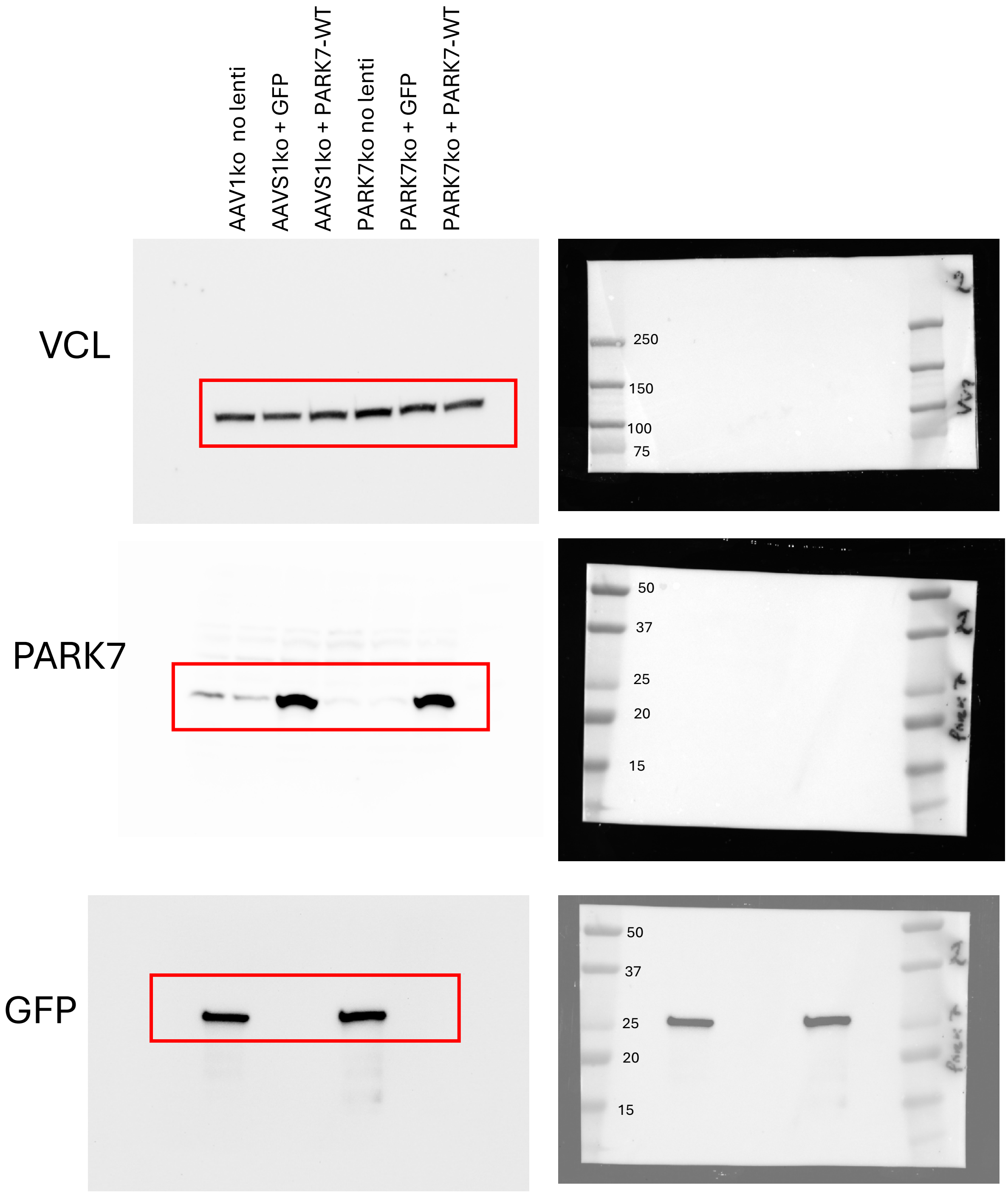


GFP after
stripping PARK7
blot

**SourceDataF4**

**4L**

PARK7ko + PARK7 WT
PARK7ko + R28*
PARK7ko + R98Q
PARK7ko + L166P

VCL

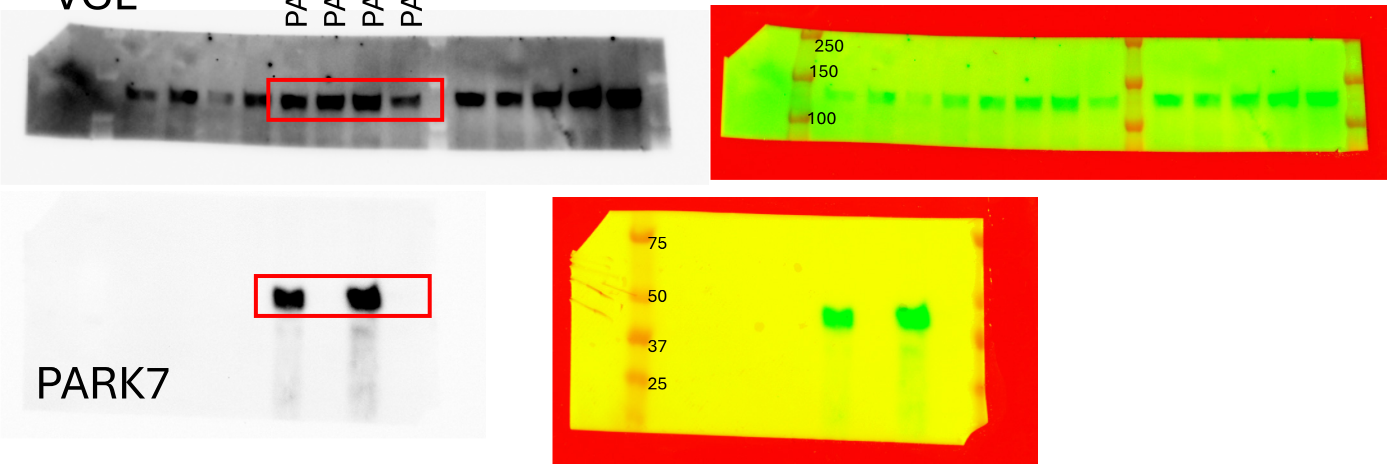


**4O**

C no lenti
C +GFP
P no lenti
P +GFP
P + PARK7 WT
P+ PARK7 R28*
P + PARK7 R98Q
P + PARK7 L166P

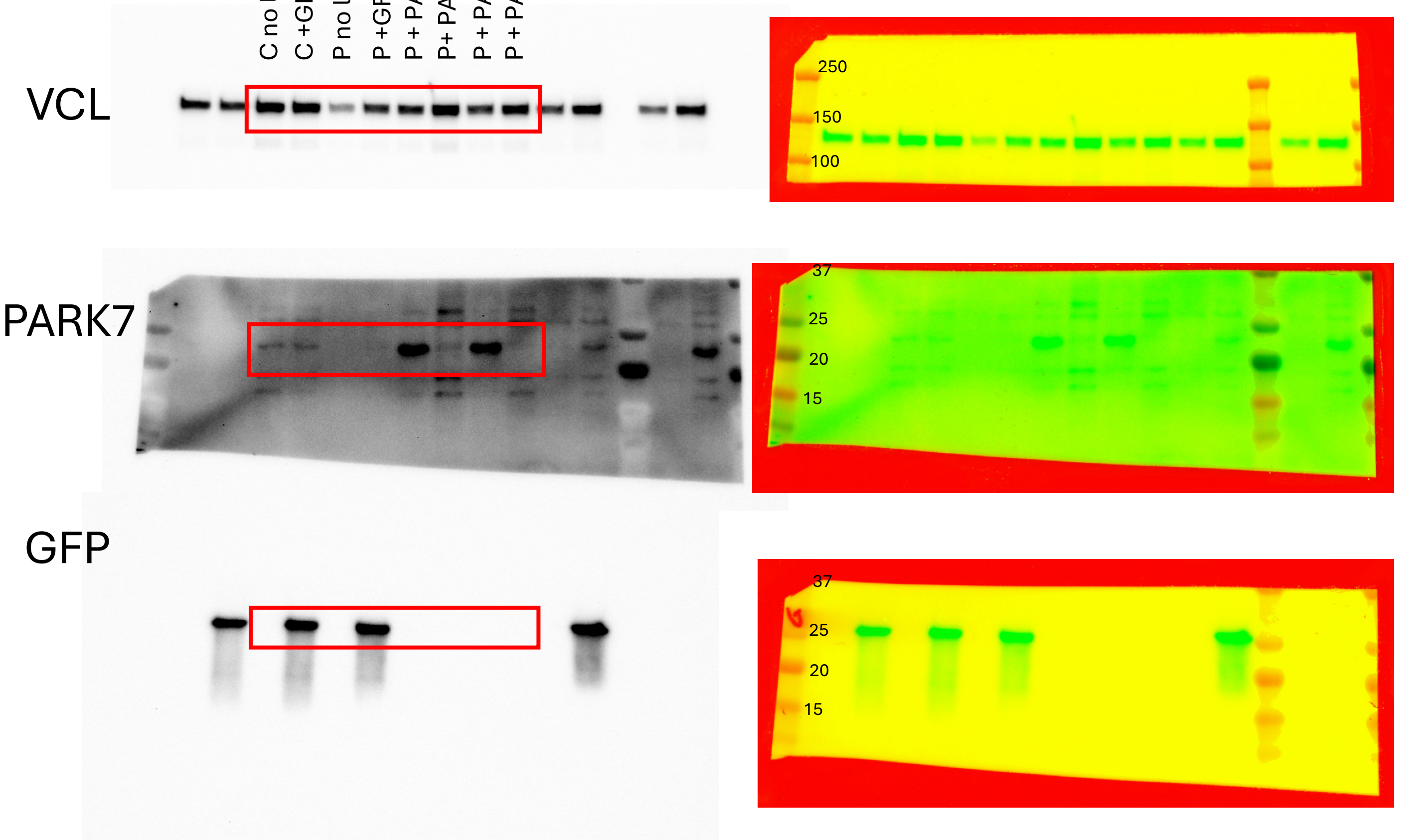


GFP after stripping PARK7 blot
